# Taxonomic Updates on the *Merodon fulcratus* and *M. spinitarsis* Species Groups, with Notes on *M. syriacus* (Diptera: Syrphidae)

**DOI:** 10.3390/insects17101078

**Published:** 2026-10-19

**Authors:** Laura Likov, Andrijana Andrić, Sanja Veselić, Iva Gorše, Snežana Radenković, Ante Vujić

**Affiliations:** 1 Department of Biology and Ecology, Faculty of Sciences, University of Novi Sad, 21000 Novi Sad, Serbia; laura.likov@dbe.uns.ac.rs (L.L.); iva.gorse@dbe.uns.ac.rs (I.G.); snezana.radenkovic@dbe.uns.ac.rs (S.R.); ante.vujic@dbe.uns.ac.rs (A.V.); 2 BioSense Institute, University of Novi Sad, 21000 Novi Sad, Serbia; andrijana.andric@biosense.rs

**Keywords:** *Merodon*, morphology, new species, hoverfly, biodiversity hotspot, distribution, *COI* gene, *28S rRNA* gene

## Abstract

Within the Palaearctic, the Anatolian and Iberian Peninsulas are established centers of diversity for the genus *Merodon* Meigen, 1803. However, recent research also highlights the Eastern Mediterranean and the Middle East as important regions. This study reviews two groups of species found across these regions, the *M. fulcratus* and *M. spinitarsis* groups, and clarifies the taxonomic status of *M. syriacus* Paramonov, 1929. The *fulcratus* species group consists of two valid species: *M. dichopticus* Stackelberg, 1968, and *M. fulcratus* (Becker, 1913). In *M. fulcratus,* high variability in the shape of the male genitalia has been observed; however, genetic analysis revealed no evidence of diversification within this species. The *spinitarsis* species group comprises *M. spinitarsis* Paramonov, 1929, and a new species from Israel: *Merodon complicatus* sp. n. This new species was previously misidentified as *M. syriacus*; however, based on the type material, *M. syriacus* proved to be closest to the East African species *M. nasicus* Bezzi 1915 from the *bombiformis* group. Furthermore, Bayesian phylogenetic analysis based on concatenated *COI* and *28S rRNA* sequences strongly supports the placement of *M. fulcratus* within *Merodon* (*Merodon*) and *M. spinitarsis* within *M.* (*Auremerodon*). This study resolves the status of several taxa and highlights important faunal links between different biogeographical regions.

## Simple Summary

A taxonomic revision of the *Merodon fulcratus* and *M. spinitarsis* species groups was carried out, including the description of one new species together with identification keys and distribution maps for the respective species. In addition, the taxonomic ambiguity surrounding the species *Merodon syriacus* was resolved. DNA analysis confirmed that *M. fulcratus* and *M. spinitarsis* belong to different evolutionary groups within the genus *Merodon*. Furthermore, despite differences in the shape of the surstylar lobe of the male genitalia, DNA analysis showed that all examined samples of *M. fulcratus* belonged to the same species.

## 1. Introduction

The hoverfly genus *Merodon* Meigen, 1803 (Diptera, Syrphidae, Merodontini), is distributed throughout the Palaearctic and Afrotropical regions, with the highest species diversity recorded in the Mediterranean Basin [1], the largest biodiversity hotspot within these regions and the second largest in the world [2]. Specifically, the northern Mediterranean has been comprehensively studied in the last decade [3,4,5,6,7,8,9,10,11,12,13,14,15], and it has been confirmed that the main center of diversity and endemism of this genus is the Anatolian Peninsula [16,17], followed by the Iberian Peninsula [18]. While the Afrotropical region and eastern Palaearctic have a low number of recorded species, the areas encompassing the north-eastern Middle East, Central Asia and Pakistan host numerous endemics of potential importance for understanding *Merodon* phylogeny [1]. An increasing number of studies in recent years have uncovered new species records and described new species from these areas [7,10,13,14,17,19,20,21,22,23], highlighting their significance for improving knowledge of *Merodon* taxonomy and distribution.

Combined analyses of morphological and molecular data carried out by Veselić et al. [24] promoted former lineages within the genus *Merodon* to subgeneric rank, establishing five subgenera: *Albimerodon*, *Auremerodon*, *Avidumerodon*, *Desutumerodon* and *Natamerodon*. In a subsequent study, currently in press, Vujić et al. further clarified the subgeneric classification within *Merodon,* with *Avidumerodon* being treated as *Merodon*, the nominotypical subgenus [25]. *Merodon* (*Merodon*) consists of 10 species groups and nine unplaced species. For most of the species groups within *M. (Merodon*) (*aberrans*, *avidus*, *aurifer*, *clavipes*, *nigritarsis*, *pruni*, *serrulatus* and *tarsatus*), taxonomic revision has already been completed [7,10,11,13,14,15,21], while for two of them (*fulcratus* and *italicus*) revisions are still in preparation. On the other hand, *M. (Auremerodon*) contains five species groups with published revisions for the *aureus* [26,27,28,29,30,31], *bombiformis* [32], and *nanus* [17,33] species groups, while the *spinitarsis* and *funestus* groups are still under revision. In addition, within *M.* (*Auremerodon*) (former *aureus* lineage), *Merodon syriacus* Paramonov, 1929, has been placed within the *aureus* species group in the parsimonious and maximum likelihood trees based on the morphological data matrix [34].

In this paper a taxonomic revision of the *fulcratus* and *spinitarsis* species groups is provided. Additionally, one new species is described, distribution maps for all analyzed species are provided, identification keys for both groups are presented, and molecular data for the *M. fulcratus* species group are included in order to clarify the observed morphological variability. Finally, the taxonomic status and systematic position of *M. syriacus* Paramonov, 1929, is clarified.

## 2. Materials and Methods

### 2.1. Morphological Analysis

The studied material is deposited in the museums and private entomological collections listed in the Abbreviations section.

The terminology used for morphological characters follows van Steenis et al. [35], except those referring to male genitalia, which follow Marcos-García et al. [18]. In order to study the male genitalia, dry specimens were relaxed in a humidity chamber. Terminalia were extracted with a hook-tipped entomological pin and boiled for 5 min in a diluted KOH solution, followed by brief immersion in acetic acid and ethanol to neutralize and remove chemical residues.

The photographs were produced with a Canon EOS 6D Mark II (Canon Inc. (headquartered in Tokyo, Japan) digital camera connected to a Canon MP-E 65MM lens Canon Inc. (headquartered in Tokyo, Japan) and processed in Helicon image stacking software 8.2.2., while for the drawings a Leica MZ16 binocular microscope (Leica Microsystems, Wetzlar, Germany) was used with an FSA 25 PE drawing tube (Leica Microsystems, Germany).

Distribution maps were generated using the mapping software QGIS 3.30.0 [36].

### 2.2. Molecular Analysis

#### 2.2.1. Laboratory Procedures

The present study is based on the DNA sequence analysis of 42 specimens of 31 species belonging to the genus *Merodon*. Information about the examined specimens is given in the Supplementary Material (Table S1). Total genomic DNA (gDNA) of specimens was extracted from the mid- and hind legs of most specimens using the NucleoSpin Tissue DNA Extraction kit (Machery-Nagel, Düren, Germany) following the manufacturer’s protocols. Each specimen used in the molecular analyses is labeled as a DNA voucher specimen. Amplifications of both the 3′-end and 5′-end of the mitochondrial *COI* gene as well as the nuclear *28S rRNA* gene were carried out in an Applied Biosystems MiniAmp Plus Thermal Cycler following the conditions described in Veselić et al. [24]. PCR amplicons were enzymatically purified using Exonuclease I and FastAP Thermosensitive Alkaline Phosphatase enzymes (ThermoScientific, Vilnius, Lithuania) according to the manufacturer’s instructions. Bidirectional sequencing was performed by the Macrogen EZ-Seq service (Macrogen Europe, Amsterdam, The Netherlands).

#### 2.2.2. Molecular Data Analyses

Chromatograms of all sequences produced for this study were edited for base-calling errors using BioEdit version 7.2.5 [37]. The alignment of both the 3′ and 5′ sequence fragments of the protein-coding *COI* gene was done manually, while the D2–3 domain of the *28S rRNA* gene was aligned using the G-INS-i strategy implemented in MAFFT version 7 [38]. The total length of the sequence by genes included in the final alignment is as follows: *COI* 1341 bp and *28S rRNA* gene 604bp, resulting in a total of 1945 nucleotide characters. GenBank accession numbers for the sequences already available in GenBank are provided for each specimen in the Supplementary Material (Table S1). The newly generated sequences have been deposited in GenBank, and the corresponding accession numbers are provided in the Supplementary Material (Table S1).

The concatenated alignments were used to infer the phylogenetic relationships using Bayesian inference. The best nucleotide substitution model for a final dataset was estimated in MEGA XI [39] and selected using the Akaike Information Criterion [40], and GTR + G + I was shown to be the most suitable for the analyzed genes. Bayesian inference trees were constructed in MrBayes 3.2.7a [41] using the CIPRES Science Gateway web portal [42]. Two independent Markov Chain Monte Carlo (MCMC) runs were performed, each with four chains, for 10 million generations. Trees and parameters were sampled every 100 generations in order to calculate posterior probabilities (PPs). Trees were visualized and rooted on *Eumerus amoenus* Loew, 1848, as an outgroup using FigTree 1.4.4 [43].

## 3. Results

### 3.1. Merodon fulcratus Species Group

**Figure 1:**
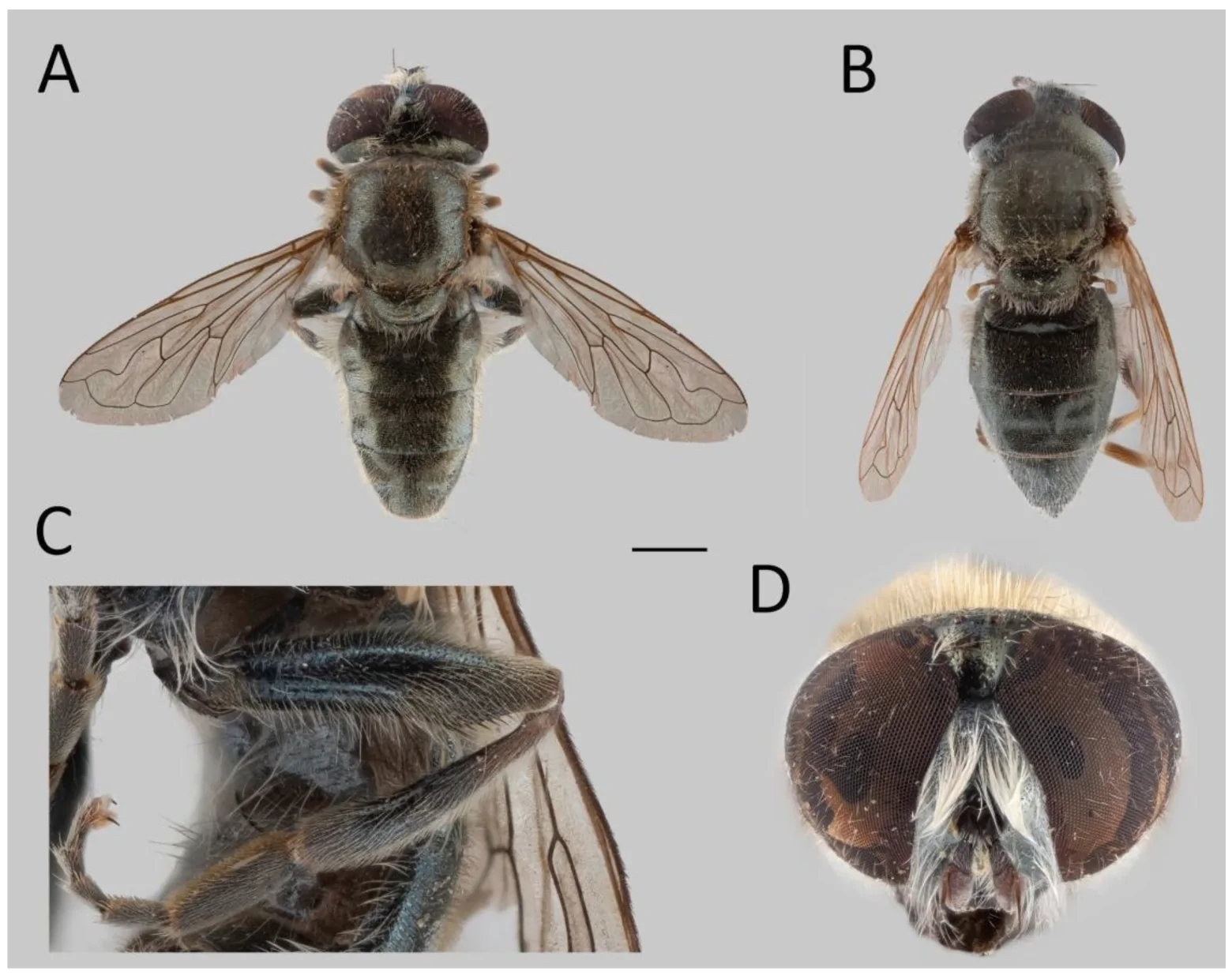
*Merodon fulcratus*. (**A**,**B**) Body, dorsal; (**C**) metaleg, anterior; (**D**) head, frontal. (**A**,**C**,**D**) Male and (**B**) female. Scale bar: (**A**,**B**) 1 mm and (**C**,**D**) 0.5 mm.

**Figure 2:**
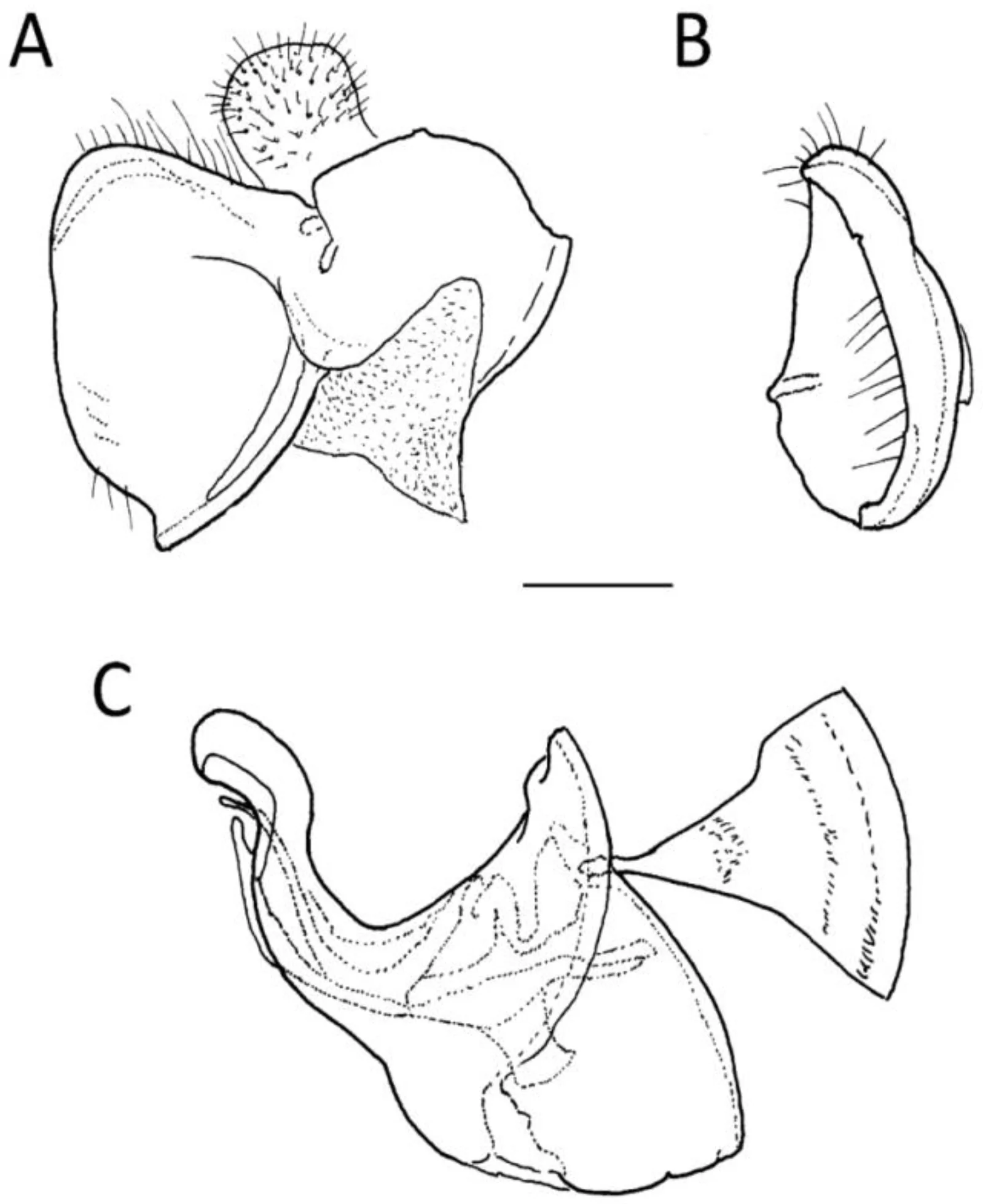
Male genitalia of *Merodon dichopticus*. (**A**) Epandrium, lateral, (**B**) surstylus, ventral, (**C**) hypandrium, lateral. Scale bar: 0.5 mm.

Diagnosis: Species from the *fulcratus* species group are small (5–9 mm) with metallic shiny bodies. The surfaces of the scutum and terga are strongly punctate (Figure 1A,B), without or with very weak pruinosity. The metafemur has a very small apicoventral femoral lamina (Figure 1C). Males of the *fulcratus* species group are clearly different from males of other species groups within *M.* (*Merodon*) by distinctly dichoptic eyes (Figure 1D) and lack of ctenidium at hypandrium (as in Figure 2C). Females cannot currently be distinguished based on morphological characters; at present, they can only be identified by molecular data. The *Merodon fulcratus* species group consists of two valid species: *M. dichopticus* Stackelberg, 1968, and *M. fulcratus* (Becker, 1913).

- *Merodon dichopticus* Stackelberg, 1968

Type material examined: HOLOTYPE: Nasirvaz, Ordubad District, Nakhichevan ASSR, 24 June 1967, ♂, V. A. Rikhter. Paratypes, 1♂, 2♀, same data as holotype [44] (ZISP).

Additional material examined: TÜRKIYE: Durnalik, 20 km W Gaziantep, 37.0833 37.3333, 13.v.2002, 1♀, Goudsmits K. leg. (FSUNS ID 03303) (RM coll.); Hakkari, Oramar, 37.3666 44.1, 1500 m asl, 16.vi.1984, 1♂ (FSUNS ID 04100) (RMNH); Durnalik, 20 km W Gaziantep, 13.v.2001, 1♂, Goudsmits K. leg. (FSUNS ID 72492) (RM coll.); Hakkari, Sat dağları (Mount Sat), Varegos, 37.4166 44.2166, 2000 m asl, 17.vi.1984, 1♀ (FSUNS ID 03302) (RMNH); Hakkari, Sat dağları (Mount Sat), Varegos, 37.4166 44.2166, 1600/1650 m asl, 18.vi.1984, 1♀ (FSUNS ID 04101), 2♂ (FSUNS ID 04164, 04163) (RMNH); Hakkari, Sat dağları (Mount Sat), Varegos, 37.4166 44.2166, 18.vi.1984, 1♂ (RMNH); Hakkari, Sat dağları (Mount Sat), Varegos, 37.4166 44.2166, 15.vi.1984, 2♂ (RMNH); Hakkari, 5 km N from Oramar, 37.3666 44.1, 16.vi.1984, 1♀ (RMNH); Kastamonu, Agli, Karacakese, 1300 m asl, 18.vi.1995, 1♀ (FSUNS ID 03313) (FSUNS); Bitlis, Tatvan, 38.5066 42.2816, 30.vi.1993, 1♀ (RMNH); ISRAEL: Mount Hermon, 32.7094 35.3158, 1700 m asl, 17.v.2009, 1♂, Friedman L. and Freidberg A. leg. (FSUNS ID 04959) (TAU); Mount Hermon, 32.7094 35.3158, 1700 m asl, 18.v.2009, 1♀, Freidberg A. leg. (TAU); Mount Hermon, 32.7094 35.3158, 1700 m asl, 17.v.2009, 3♂ 4♀, Friedman L. and Freidberg A. leg. (TAU); Mount Hermon, 32.709474 35.315874, 1600 m asl, 04.v.1999, 1♂, Friedman L. leg. (TAU); Mount Hebron, 32.7094 35.3158, 1600 m asl, 23.v.1998, 1♀, Freidberg A. leg. (FSUNS ID 04960) (TAU); Hebron desert, 26.iii.1974, 1♀, det. by Hurkmans W. as *M. nanus* (TAU); GEORGIA: Tbilisi, vi.1957, 1♀, Maran J. leg. (FSUNS ID 18244) (NMPC); IRAQ: Kurdistan, Haji Ohuran, 5500 ft (1676 m asl), 07–08.vi.1957, 1♂, det. by Hurkmans as *Merodon fulcratus* (BMNH); UNK. COUNTRY, no data, 1♀ (FSUNS ID 03304) (ZHMB).

Type locality: For a type locality of *M. dichopticus* Evenhuis & Pape (2025) [45] listed as Russia, South Caucasia, Nakhitschev. Based on the original description of the species, the type material is from Nasirvaz, Ordubad District, Nakhichevan ASSR, 24 June 1967 [44]. The mentioned district and city are part of Nakhchivan Autonomous Republic, a true exclave of Azerbaijan.

**Figure 3:**
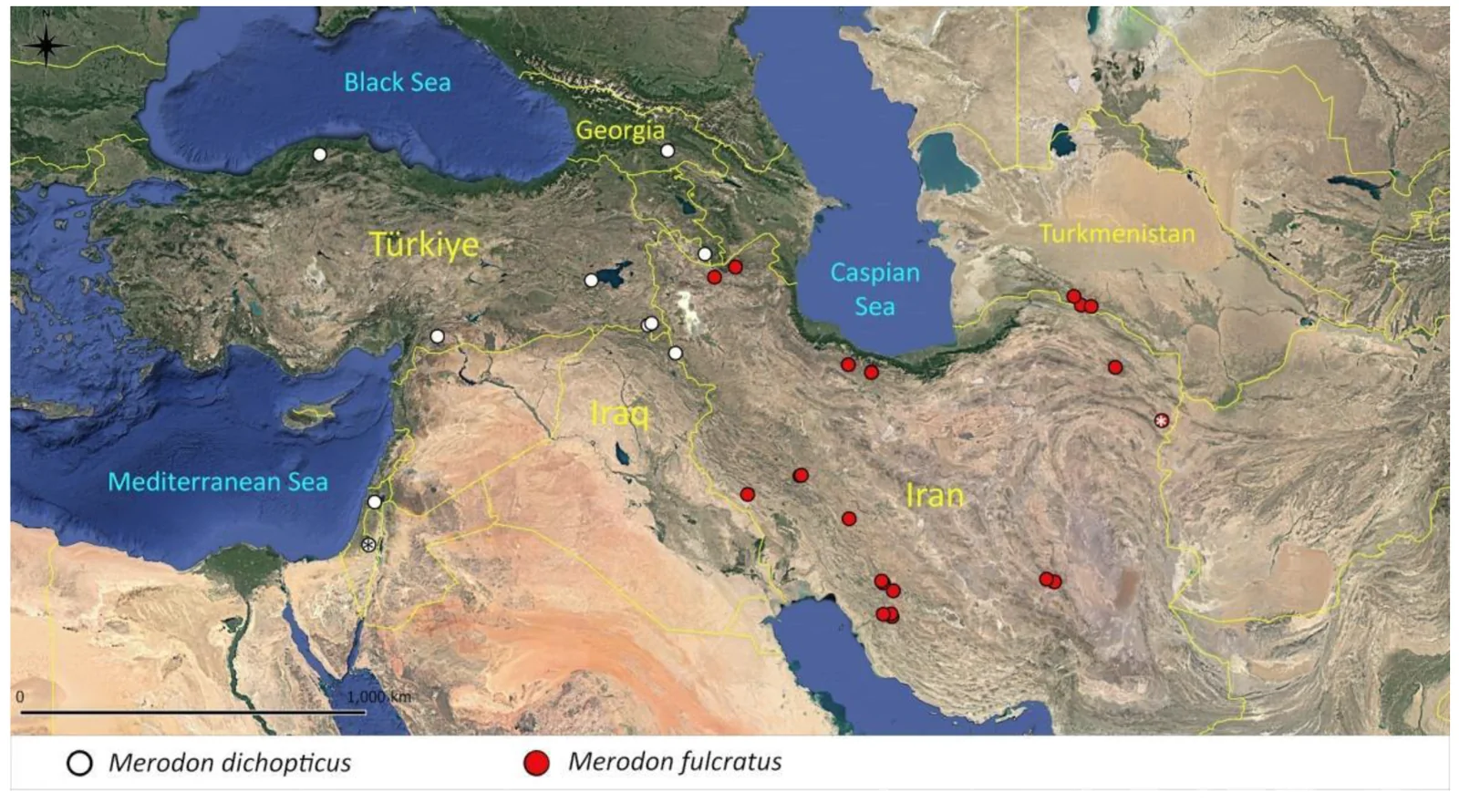
Distribution map of the *Merodon fulcratus* species group. A white dot with an asterisk indicates the presence of *M. dichopticus* on Hebron Mountain and in Hebron desert, but without exact coordinates available; a red dot with an asterisk indicates the approximate location of the *M. fulcratus* syntypes, but without information about exact coordinates.

Distribution: The distribution area of *M. dichopticus* includes parts of Azerbaijan, Türkiye, Israel, Georgia and Iraq (Figure 3).

**Figure 4:**
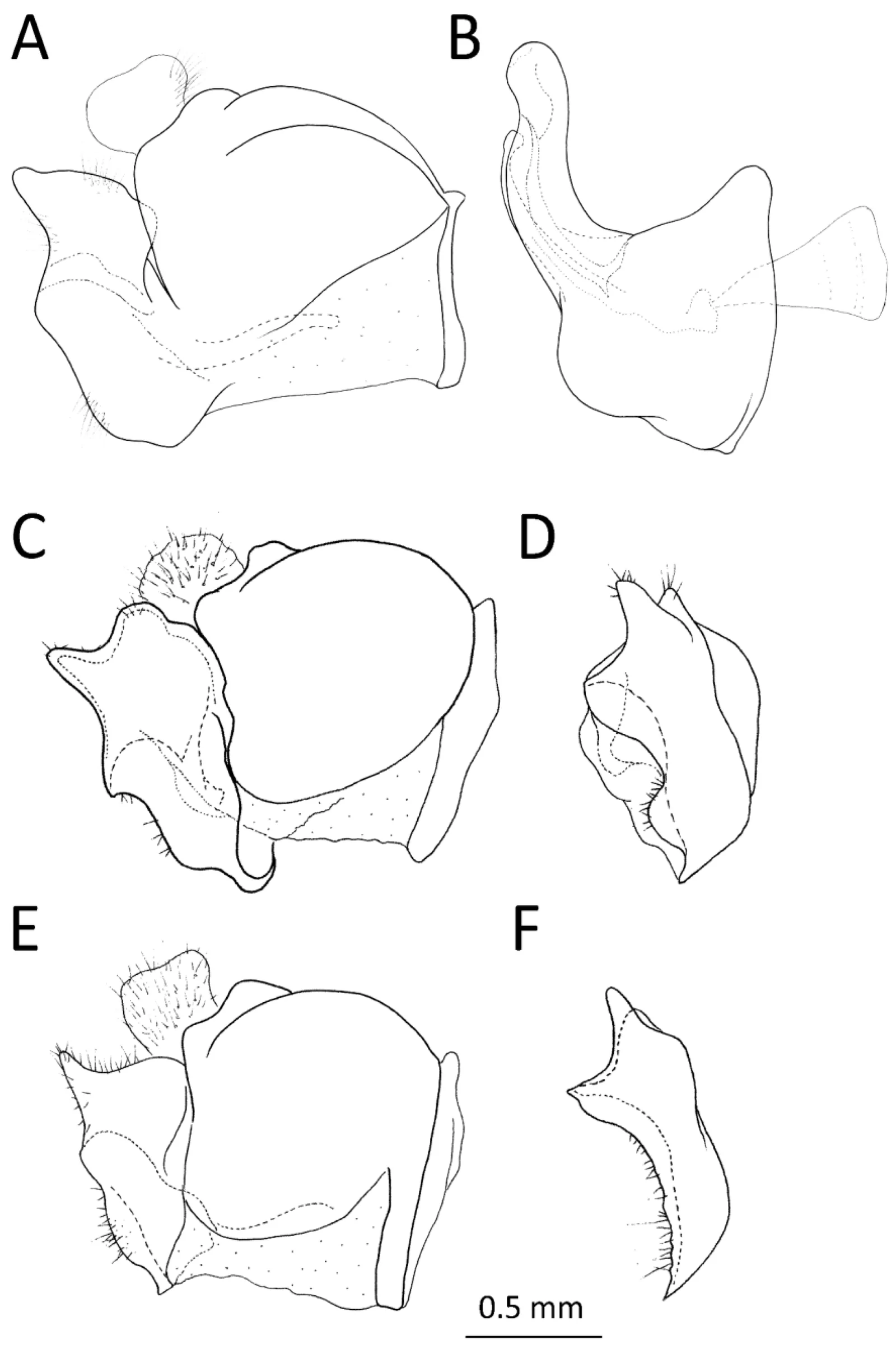
Male genitalia of *Merodon fulcratus*. (**A**,**C**,**E**) Epandrium, lateral, (**B**) hypandrium, lateral, (**D**,**F**) surstylus, ventral. Scale bar: 0.5 mm.

Diagnosis: Males of two species in the *fulcratus* species group can be easily distinguished based on the structure of genitalia: apex of the posterior surstylar lobe is rounded and ventral margin straight in *M. dichopticus* (Figure 2A), while in *M. fulcratus* apex is tapering and ventral margin wavy (Figure 4A,C,E).

- *Merodon fulcratus* (Becker, 1913)

*Exmerodon fulcratus* in Becker, 1913 (604), Figure 28 in [46].
Lampetia brandti by Doesburg (unpublished).

Note: Becker’s collection is not deposited in a single museum. The vast majority of Becker’s personal insect collection and primary name-bearing type series were deposited in the Museum für Naturkunde (MNB) in Berlin, the Zoological Institute of the Russian Academy of Sciences in St. Petersburg (ZISP), and other regional museums. The author (A.V.) who studied the type material and visited these two main museums as well as many other European museums traced many of Becker’s types, but not this one. Curators at the museums were unable to provide any information regarding the potential repository of the collection. Consequently, the syntypes (two males: Iran, Chorassan, Gerri-rud, Landschaft Bechars. 8–10. IV. 1898.) [46] are presumed to be lost, which was the main reason for proposing the designation of a neotype. As demonstrated by our results, *Merodon fulcratus* is widely distributed throughout Iran. The designation of the neotype was based on a well-preserved specimen displaying all key morphological characters being consistent with what is known of the former name-bearing type from the original description [47]. It did not originate from the exact type locality but from the broader type area, which is nearly as practicable. The above facts are in accordance with Article 75 of the International Code of Zoological Nomenclature.

Part of the museum material was labeled as Lampetia brandti by Doesburg. This name was never formally published; however, specimens bearing this label can still be found in some collections.

NEOTYPE (designated here): IRAN, Lorestan, Dorud, Lanjabad, 33.433 49.029, 670 m asl, 11.v.2016, 1♂, Kafka M. leg. (FSUNS ID 72262) (FSUNS).

Examined material: IRAN, Khorasan Razavi, Shandiz Zoshk, 36.2825 59.1197, 27.v.2006, 1♀, Gilasian leg. (03312) (FSUNS); Tehran, 6 km N Gacsa, 36.15 51.283, 1.vi.2002, 1♀, Prochazka leg. (03309) (BM coll.); Kerman, Darbe Asiab, 30.5203 57.1617,01.v.2007, 1♀, Gilasian and Nematian leg. (03942) (FSUNS); Tehran, 6 km N Gacsa, 36.15 51.28333, 1.vi.2002, unk. sex, Prochazka leg. (72169) (unk. coll.); Qaradagh forests, 38.843067 46.9118, 2♂, Khaganinia leg. (10290, DNA AU1143) (10289, DNA AU1144) (KS coll.); Juladak, Moalem Kelayeh, Almot Ghazvin province, 36.351944 50.53833, 2300–250 0m asl, 30.v–9.vii.2007, 2♂, Babak Gharali leg. (05790, 05789) (unk. coll.); Fars, Sepidan, 30.2722 51.9844, 08.v.2007, 1♂, Gilasian and Nematian leg. (03940) (FSUNS); Tehran, 6 km N Gacsa, 36.15 51.283, 1.vi.2002, 1♀, Prochazka leg. (03307) (BM coll.); Fars, 15 km S of Dasht Arjan, 29.5523806 51.9395056, 2261 m asl, 2–6.v.2016, 1♀, Martin Oboril leg. (18274) (HJ coll.); Lorestan prov., Dorud, Lanjabad, 33.419 48.986, 950 m asl, 10.v.2016, 3♂, Kafka M. leg. (72175, 72170, 72172) (unk. coll.); Fars prov., Yasuj Sarab-e Taveh, 30.49 51.658, 2390 m asl, 4.v.2016, 3♂, Kafka M. leg. (72174, 72171, 72173) (unk. coll.); Fars prov. Yasuj, Sarb-e-Taveh, 30.545 51.61, 2030 m asl, 04.v.2016, 1♂, Kafka M. leg. (FSUNS ID 72265, DNA ID TS836) (FSUNS); Lorestan, Lanjabad, 33.419 48.986, 950 m asl, 10.v.2016, 2♂, Kafka M. leg. (FSUNS ID 72260, 72261) (FSUNS); Fars, Dasht Ajran, 29.63 51.912, 2040 m asl, 05.v.2016, 3♂, Kafka M. leg. (FSUNS ID 72255, 72256, 72257) (FSUNS); Lorestan, Lanjabad, 33.433 49.029, 1670 m asl, 10.v.2016, 1♂, Kafka M. leg. (FSUNS ID 72258) (FSUNS); Ilam prov., Dinar Kouh, 32.915 47.301, 1830 m asl, 12.v.2016, 1♂, Kafka M. leg. (FSUNS ID 72259) (FSUNS); Fars prov. Yasuj, Sarb-e-Taveh, 30.545 51.61, 2030 m asl, 04.v.2016, 3♂, Kafka M. leg. (FSUNS ID 72263, 72264, 72272) (FSUNS); Fars, Sepidan, 30.2722 51.9844, 2400 m asl, 25.iv.2006, 1♀ (03311) (FSUNS); SW Isfahan, Farsan, 32.2556 50.5641, 12.v.1992, 1♂ (RMNH); Fars, Sepidan, 30.5203 57.1617, 2640 m asl, 01.v.2007, 1♂ (03941) (FSUSN); no data (03301) (FSUSN); Chichekli forest, 38.588067 46.239583, 1♂, Khaganinia leg. (10295) (KS coll.); Fars, Rd Chiraz Kazeroun, Fort Sine-Sefid, 29.61833 51.65833, 23.iv.1937, 1♀, det. by P.H. van Doesburg as Lampetia brandti (03310) (RMNH); Kerman-chatroud, sardar, 30.59805 56.9075, 555 m asl, 06.v.2013, 1♂ 1♀, Khosravijan Chatroodi Zoerh leg. (05860, 05859) (KCZ coll.); Fars rd Chiraz-Kazeroun, Fort Sine-Sefid, 28.iv.1937, 1♂ 1♀, Brandt F. H. leg., det. by P. H. van Doesburg as Lampetia brandti (RMNH); Fars rd Chiraz-Kazeroun, Fort Mian-Kotal, 25.iv.1937, 1♂, Brandt F. H. leg., det. by P. H. van Doesburg as Lampetia brandti (RMNH); Fars rd Chiraz-Kazeroun, Fort Sine-Sefid, 25.iv.1937, 1♂, Brandt F. H. leg., det. by P. H. van Doesburg as Lampetia brandti (RMNH); Fars rd Chiraz-Kazeroun, Fort Sine-Sefid, 23.iv.1937, 1♂, Brandt F. H. leg., det. by P. H. van Doesburg as Lampetia brandti (RMNH); Tehran, Gacsa, 36.15 51.2833, 01.vi.2002, 1♀ (03308) (BM coll.); Fars rd Chiraz-Kazeroun, Fort Sine-Sefid, 29.6183 51.6583, 29.iv.1937, 2♂, det. by P. H. van Doesburg as Lampetia brandti (03306, 03305) (RMNH); TURKMENISTAN, Okr. Anhabada pos. Firjuza [oкp. Aнxaбaдa, пoc. Φиpюзa], 17.iv.1997, 1♂, Barkalov Anatolij leg. (05850) (SZMN); 20 km jujuv Annaj [20 км ююв Aннay], 29.iv.1988, 1♂, Barkalov Anatolij leg. (05851) (SZMN); SSR, 15 km zap. Firjuza [CCP. 15 км зaп. Φиpюзa], g. Dušak [г. Дyшaк], 16.iv.1988, 2♀, Barkalov Anatolij leg. (05852, 05853) (SZMN); Kopetdag, okr. Čuli [Koпeтдaг, oкp. Чyли], 05.v.1984, 1♀, leg. Ozerov A. (25357) (SIZK); SSR, 15 km zap. Firjuza [15 км зaп. Φиpюзa], g. Dušak [г. Дyшaк], 08.iv.1988, 1♂, Barkalov Anatolij leg. (05854) (SZMN).

Type area: IRAN: Chorassan, Behars, Gerri-rud (Becker, 1913).

Distribution: *Merodon fulcratus* is recorded throughout Iran and in southern Turkmenistan (Figure 3).

Diagnosis: In *M. fulcratus* a significant variability in the shape of male genitalia, precisely in the posterior surstylar lobe, has been observed (Figure 4A,C,E). However, genetic analysis showed no evidence of diversification within this taxon. Males in the *fulcratus* species group can be easily distinguished based on the structure of male genitalia: apex of the posterior surstylar lobe is tapering in *M. fulcratus* (Figure 4A,C,E), while it is rounded in *M. dichopticus* (Figure 2A).

Key to the Species of the *Merodon fulcratus* Species Group

1. Male genitalia: Posterior surstylar lobe rounded with a straight ventral margin (Figure 2A) …*M. dichopticus*

- Male genitalia: Posterior surstylar lobe tapering with a wavy ventral margin (Figure 4A,C,E)…*M. fulcratus*

### 3.2. Merodon spinitarsis Species Group

**Figure 5:**
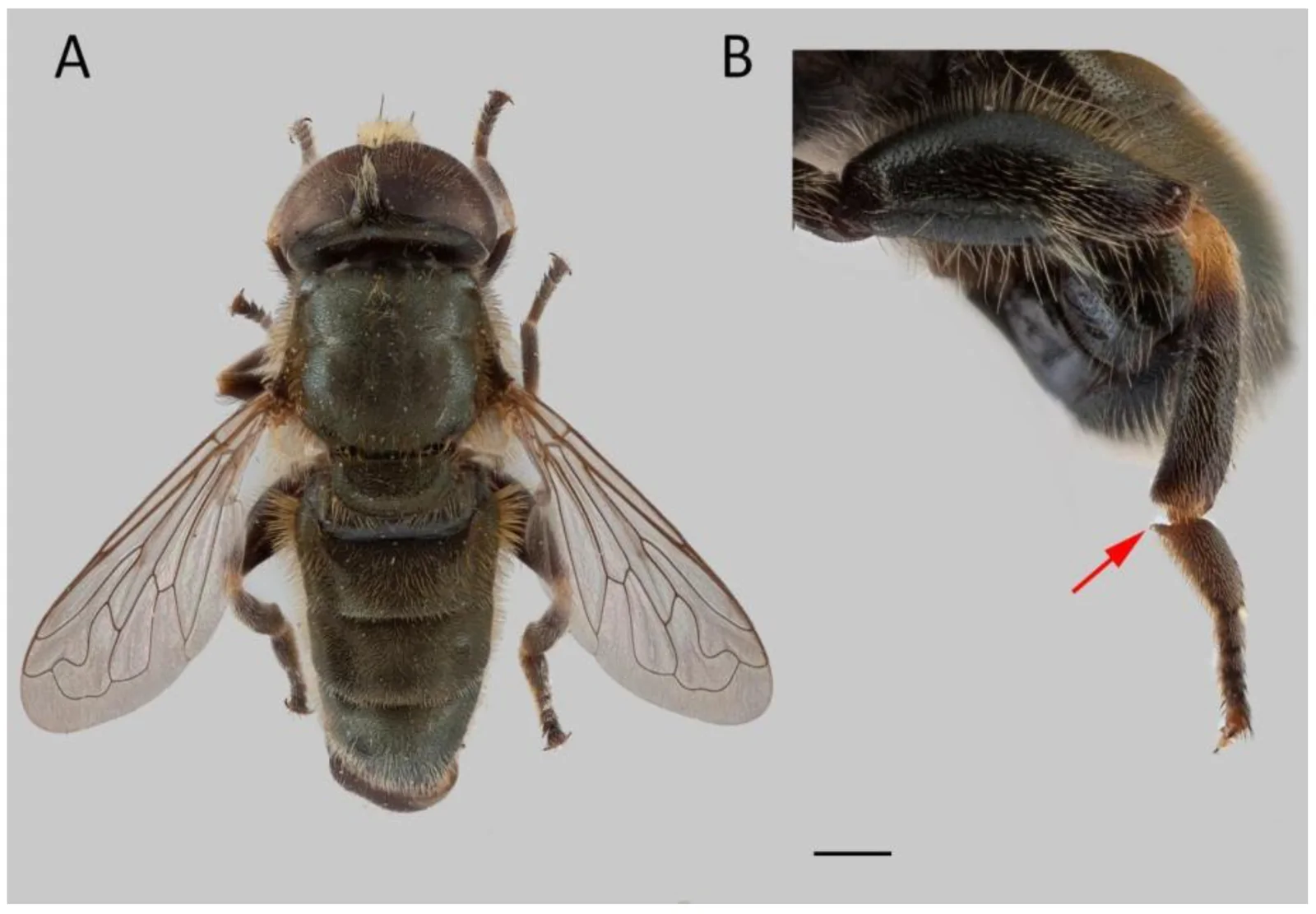
Male of *Merodon spinitarsis*. (**A**) Body, dorsal, and (**B**) metaleg, anterior. Scale bar: (**A**) 2 mm and (**B**) 1 mm. The red arrow points to the basoventral lamina of the basitarsomere of the metaleg.

**Figure 6:**
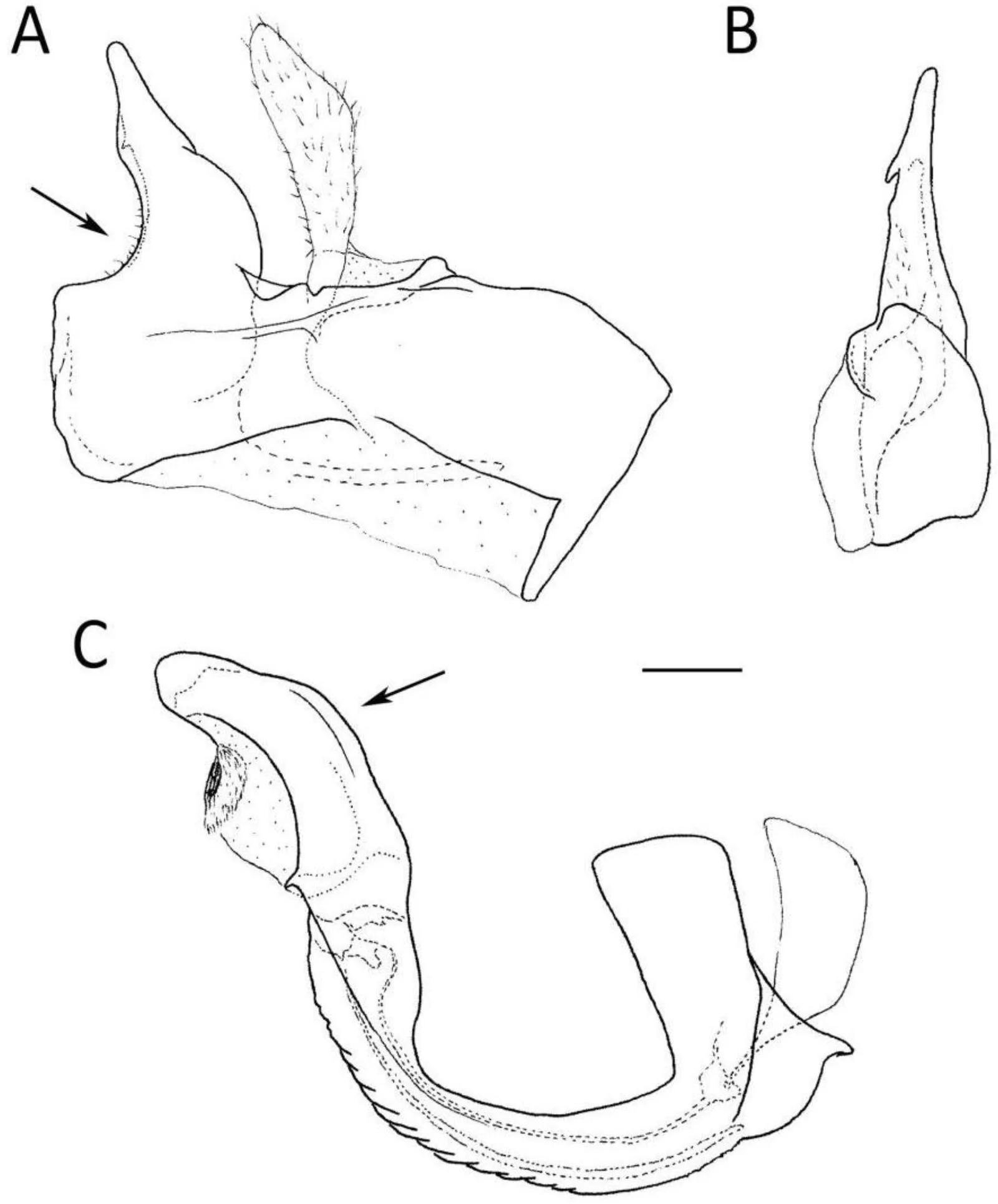
Male genitalia of *Merodon spinitarsis* (FSUNS ID 07975). (**A**) Epandrium, lateral; (**B**) surstylus, ventral; (**C**) hypandrium, lateral. Scale bar: 0.5 mm.

**Figure 7:**
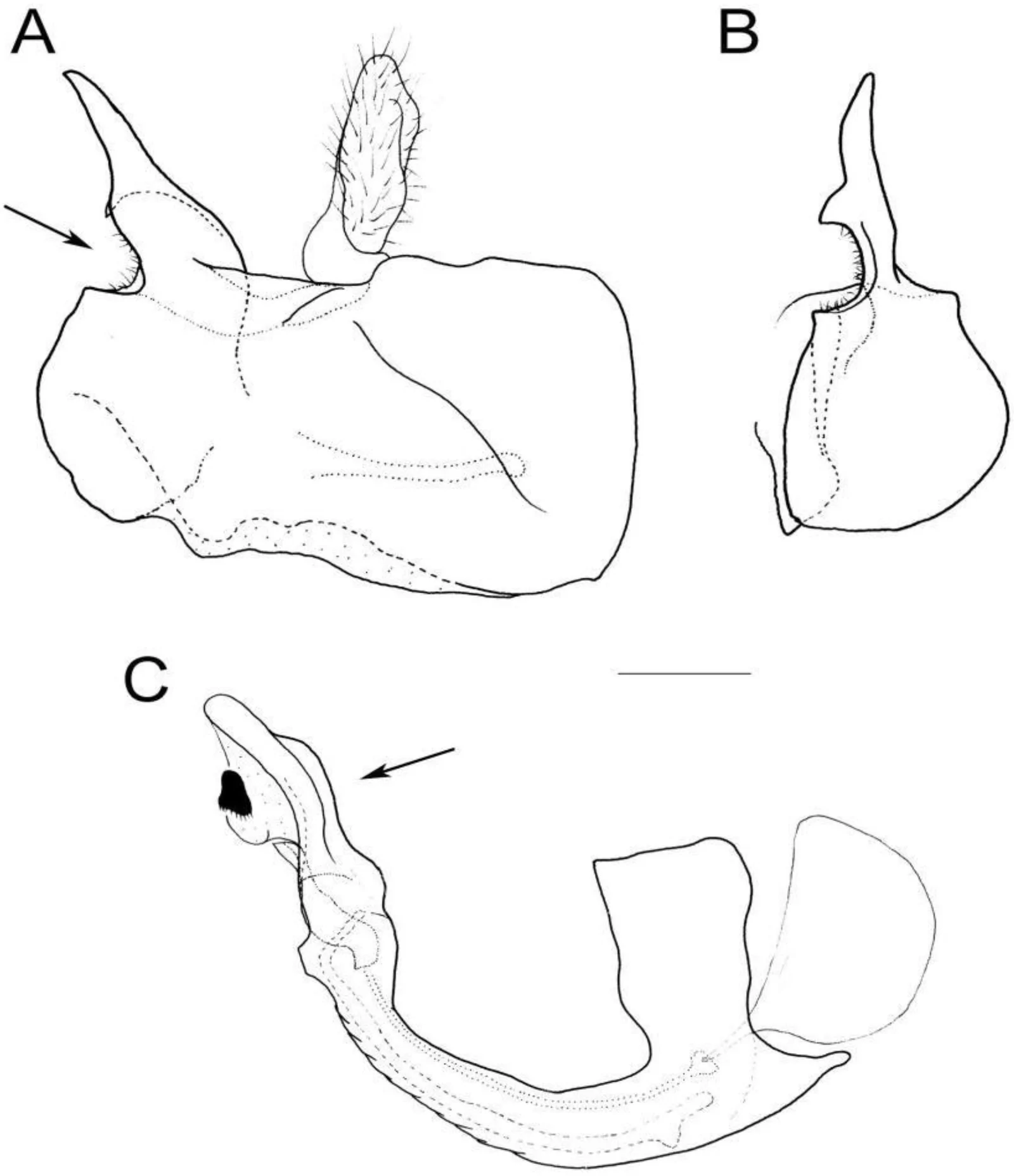
Male genitalia of *Merodon complicatus* sp. n. (paratype) (FSUNS ID 03326). (**A**) Epandrium, lateral; (**B**) surstylus, ventral; (**C**) hypandrium, lateral. Scale bar: 0.5 mm.

**Figure 8:**
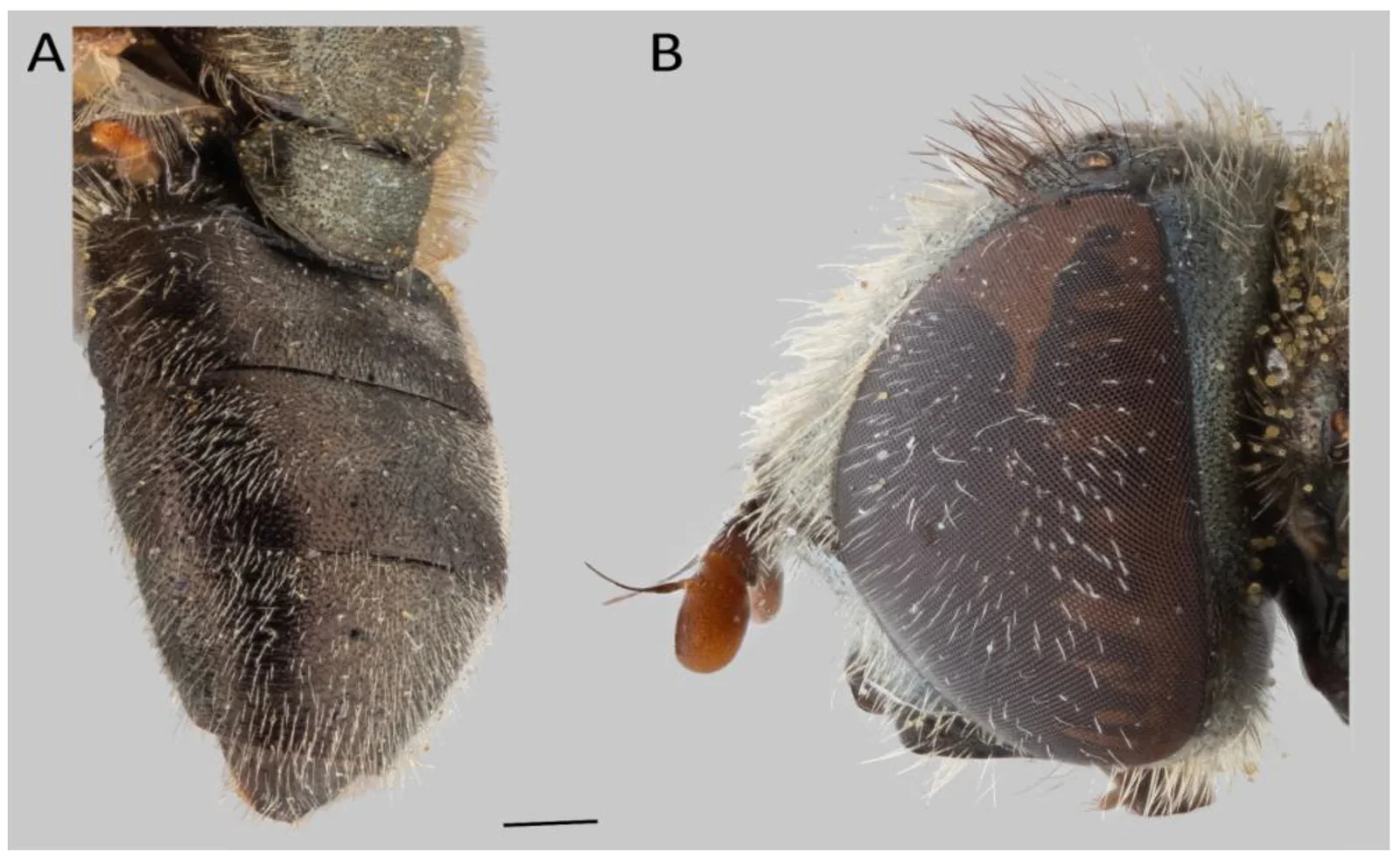
Female of *Merodon complicatus* sp. n. (**A**) Abdomen, dorso-lateral; (**B**) head, lateral. Scale bar: 0.5 mm.

Dark brown-olive species without pruinosity on terga, which are densely punctured and covered with short, adpressed pili, the metatrochanter rounded and smooth, and the tibiae (except yellowish base) and tarsi predominantly black (Figure 5B). Males of this species group characterized unique structure of the male genitalia: strongly modified hypandrium, sinuous in apical half, with subapical ctenidium and stitched theca (Figure 6C), and narrow and pointed posterior surstylar lobe (Figure 6A), with some variability in the prominence of the concavity at the margin between anterior and posterior surstylar lobes (Figure 6A and Figure 7A: marked with arrow) and in the width of the apical part of theca of the hypandrium (Figure 6C and Figure 7C: marked with arrow) (both forms are found in each of the two species belonging to this species group). On the other hand, the pili on terga in females are shorter and more adpressed as in males (Figure 8A). The *Merodon spinitarsis* species group, belonging to *M.* (*Auremerodon*), comprises two species: *M. spinitarsis* Paramonov, 1929, from Bulgaria, Romania, Greece and Türkiye, and *Merodon complicatus* sp. n. from Israel, which has so far been incorrectly listed as *M. syriacus*.

- *Merodon spinitarsis* Paramonov, 1929

Examined material: BULGARIA, 1♀; GREECE, 305 ♂, 177 ♀; TÜRKIYE, 120 ♂, 65 ♀; ROMANIA, 1 unk. sex. Due to the large volume of examined material, the details of each specimen are found in the Excel table in the Supplementary Material (Table S2).

**Figure 9:**
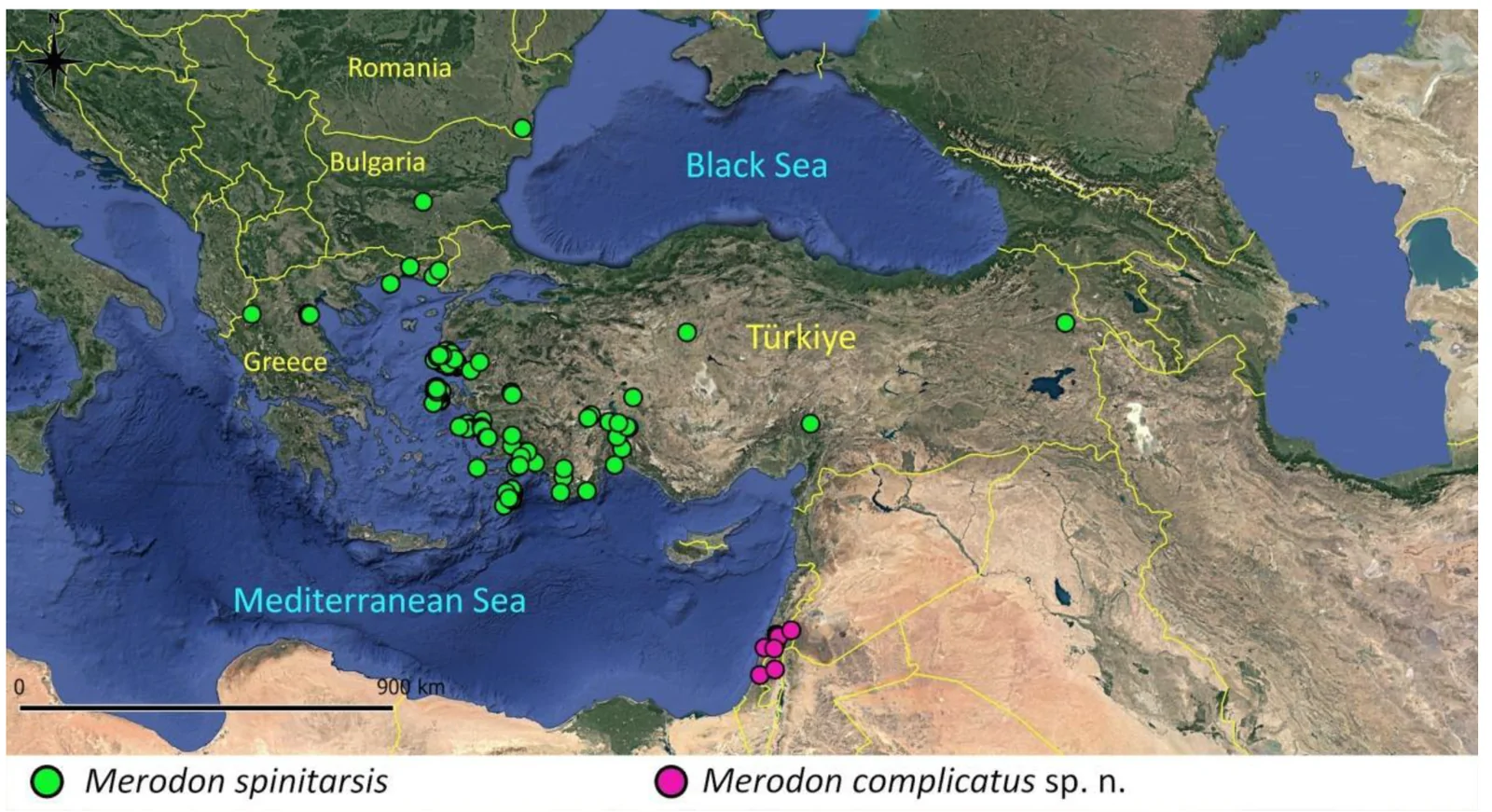
Distribution map of *Merodon spinitarsis* species group.

Distribution: This species is registered in some regions of Bulgaria, Romania, Greece and Türkiye (Figure 9).

Diagnosis: *Merodon spinitarsis* is small (5–10 mm), dark brown-olive species without pruinosity on terga, which are densely punctured, covered with short, adpressed pili (Figure 5A) (shorter and more adpressed in females), the tibiae and tarsi are predominantly black, and the basitarsomere of metaleg has basoventral lamina (present only in males) (Figure 5B). Male genitalia are very characteristic: the posterior surstylar lobe is elongated and pointed (Figure 6A), and the hypandrium is strongly modified (Figure 6B). The only characteristic to distinguish this species from *M. complicatus* sp. n. is the presence of the basoventral lamina on the basitarsomere of metaleg in *M. spinitarsis* (Figure 5A). Females are currently distinguished from *M. complicatus* sp. n. only on the basis of distribution (Figure 9).

- *Merodon complicatus* sp. n. Vujić, Likov et Radenković

urn:lsid:zoobank.org:act:008CA922-F08A-46B0-A1B8-56032C1399C9 

Examined material:

Holotype: ISRAEL, Mt. Meiron, 1100 m asl, 30.iv.1981, 1♂, A. Freidberg leg., as *M. nanus* by Hurkmans (FSUNS ID 04915) (TAU).

Paratypes: ISRAEL, Sasa, 27.iv.1984, 1♀, A. Freidberg leg., det. as *M. nanus* by Hurkmans in 1993 (FSUNS ID 04961) (TAU); Har Karmel (Mt. Haifa), 32.7286111 35.0466667, 16.iv.1980, 1♀, J.A.W. Lucas leg. (FSUNS ID 03327) (RMNH); Har Meron reserve, en Zeved, 32.9833 35.4333, 24.iv.2002, 1♂, Freidberg A. leg. (FSUNS ID 04896) (TAU); Har Karmel (M. Haifa), 32.7286111 35.0466667, 16.iv.1980, 1♀, J.A.W. Lucas leg. (FSUNS ID 03325) (RMNH); Mt. Meiron, 1100 m asl, 27.iv.1984, 1♀, Freidberg A. leg. (FSUNS ID 04941) (TAU); Meiron, 32.983333 35.433333, 13.v.1973, 1♀, Nachbar F. leg., det. as *M. nanus* by Hurkmans in 1993 (FSUNS ID 04962) (TAU); Petah Tiqwa, 30.iii., 1♂, Theodor O. leg. (TAU); Mt. Meiron, 27.iv.1984, 1♂, Freidberg A. leg., det. as *M. syriacus* by Hurkmans W. (TAU); Mt. Meiron, 1100 m asl, 27.iv.1984, 1♂, Freidberg A. leg., det. as *M. syriacus* by Hurkmans W. (TAU); Sasa, 27.iv.1984, 1♀, A. Freidberg leg., det. as *M. nanus* by Hurkmans in 1993 (TAU); Haifa Carmel forest, 33.1333, 35.7833, 25.iii.1984, 1♂, O`Toole C. leg., det. as *M. syriacus* by Hurkmans W. (TAU); Har Meron reserve, en Zeved, 32.9833 35.4333, 24.iv.2002, 3♂ 6♀, Freidberg A. and Friedman L. leg. (TAU); Mount Hermon, 32.709474 35.315874, 1500–1650 m asl, 22.v.1990, 1♂, Freidberg A. leg. (TAU); Meron, 13.v.1973, 1♀, Kaplan M. leg., det. as *M. nanus* by Hurkmans (TAU); Meron Golan, 33.1333 35.7833, 09.v.2006, 1♀, Afik R. leg. (TAU); Carmel, 05.v.1976, 1♀, Freidberg A. leg., det. as *M. nanus* by Hurkmans (TAU); Meron, 05.v.1975, 1♂, det. as *M. syriacus* by Hurkmans (TAU); West Bank, Elon, 05.v.19??, 1♀, Bylinski Salz leg. (TAU); UNK. COUNTRY, 1♂, det. by Vujić as *M. syriacus* in 2005 (FSUNS ID 03326) (RMNH).

**Figure 10:**
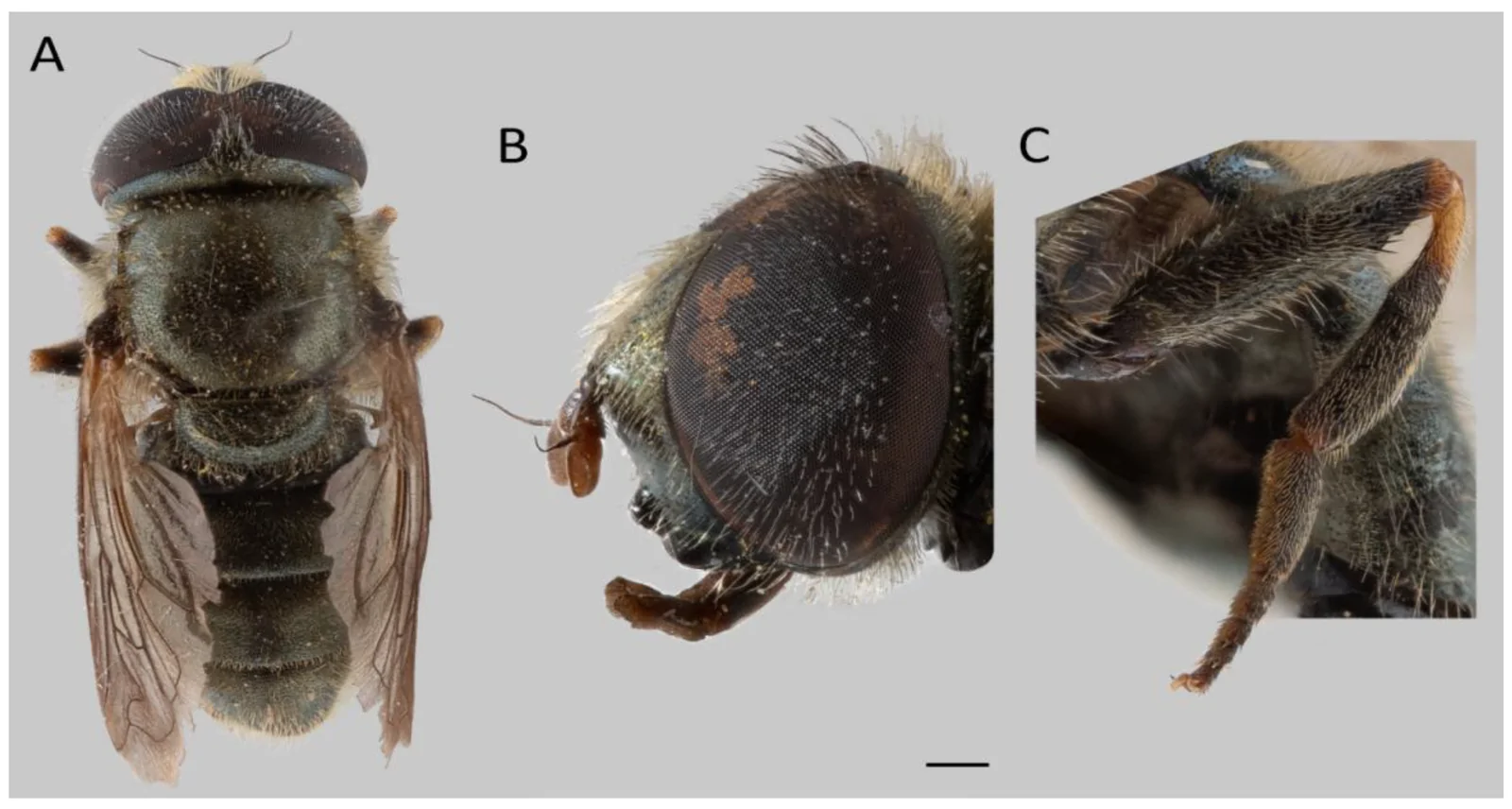
Male of *Merodon complicatus* sp. n. (**A**) Body, dorsal; (**B**) head, lateral; (**C**) metaleg, anterior. Scale bar: (**A**) 1 mm and (**B**,**C**) 0.5 mm.

Diagnosis: *Merodon complicatus* sp. n. is a small (8–10 mm), dark brown-olive species, with strongly punctured cuticle and predominantly black legs. The posterior surstylar lobe of male genitalia is elongated and pointed (Figure 7A), while the hypandrium is sinuous in apical half and with stitched theca (Figure 7C). In general appearance is very similar to *M. spinitarsis*, with only one difference: in *M. spinitarsis* a basoventral lamina is present on basitarsomere of metaleg (Figure 5B), which is absent in *M. complicatus* sp. n. (Figure 10C). Females are currently distinguished from *M. spinitarsis* only on the basis of distribution: *M. complicatus* sp. n. is so far registered only in Israel (Figure 9).

Description:

Length: Body 8–10 mm and wing 4–6 mm (*n* = 8).

Male. Head (Figure 10B): The antenna is brown, the basoflagellomere is about 1.5–1.6 times as long as wide; the arista is relatively short, slightly longer than the basoflagellomere. The face and frons are black, shiny (pruinosity is indistinct and sparse), covered with long whitish-yellow pili. The oral margin is shiny black, well protruded. The vertical triangle is isosceles, shining black, covered predominantly with pale yellow pili, except a few black pili in the anterior half; the ocellar triangle is equilateral. The eye contiguity is about 10–12 facets long. The occiput is black with grayish sheen and narrow, grayish pruinosity along the eye margin, covered with pale yellow pili. The eyes are covered with whitish pili.

Thorax: The mesonotum is black with dark golden-olive sheen (pruinosity is indistinct), densely covered with erect yellow pili. The pleura are partly covered with gray pruinosity. The proepimeron, posterior anepisternum, anteroventral and posterodorsal parts of katepisternum, anepimeron, metasternum and katatergum are covered with long yellow pili. The wing is hyaline, completely covered with microtrichia; veins are yellow-brown. The calypter is bright yellow with dense long, pale yellow marginal pili. The haltere is yellowish-brown (darker below the capitulum). The legs are predominantly black, except paler apex of all femora, base of tibiae, and tarsi basoventrally that are slightly orange-yellow. The metafemur is moderately thick, about 3 times as long as wide (Figure 10C). The pili on legs are predominantly yellow, except some black pili dorsally on pro- and mesofemora, and some strong black setae on tarsi.

Abdomen (Figure 10A): The abdomen is broad and short, as long as mesonotum. The terga are black, with dark golden-olive sheen, strongly punctured, and covered with semi-erect and adpressed golden-yellow hairs. The sterna are brownish-black, shiny, covered with pale pili.

Male genitalia (Figure 7): Anterior surstylar lobe is absent; the posterior surstylar lobe is elongated, narrow and pointed; the cercus is elongated, narrow and rectangular. Hypandrium is strongly modified, sinuous in apical half, with subapical ctenidium and stitched theca (cuticle looks like it is sewed).

Female (Figure 8): Except normal sexual dimorphism, in females the basoflagellomere is more elongated than in males, about 1.7–1.8 longer than wide, and the pili on terga are more adpressed and sparse (Figure 8A).

Distribution: So far, this species has only been recorded in Israel (Figure 9).

Etymology: The Latin adjective *complicatus* (“complicated”), referring to the complex history of misinterpretation and the use of different names for this taxon in collections and publications.

Key to the Species of the *Merodon spinitarsis* Species Group

1. Basoventral lamina on the basitarsomere of metaleg present (Figure 5A); females are currently separated only on the basis of distribution (Bulgaria, Greece, Romania and Türkiye (Figure 9))… *M. spinitarsis*

- Basoventral lamina on the basitarsomere of metaleg absent (Figure 10C); females are currently separated only on the basis of distribution (Israel (Figure 9)) …*M. complicatus* sp. n.

### 3.3. Nomenclatural Clarification Within Merodon (Auremerodon)

**Figure 11:**
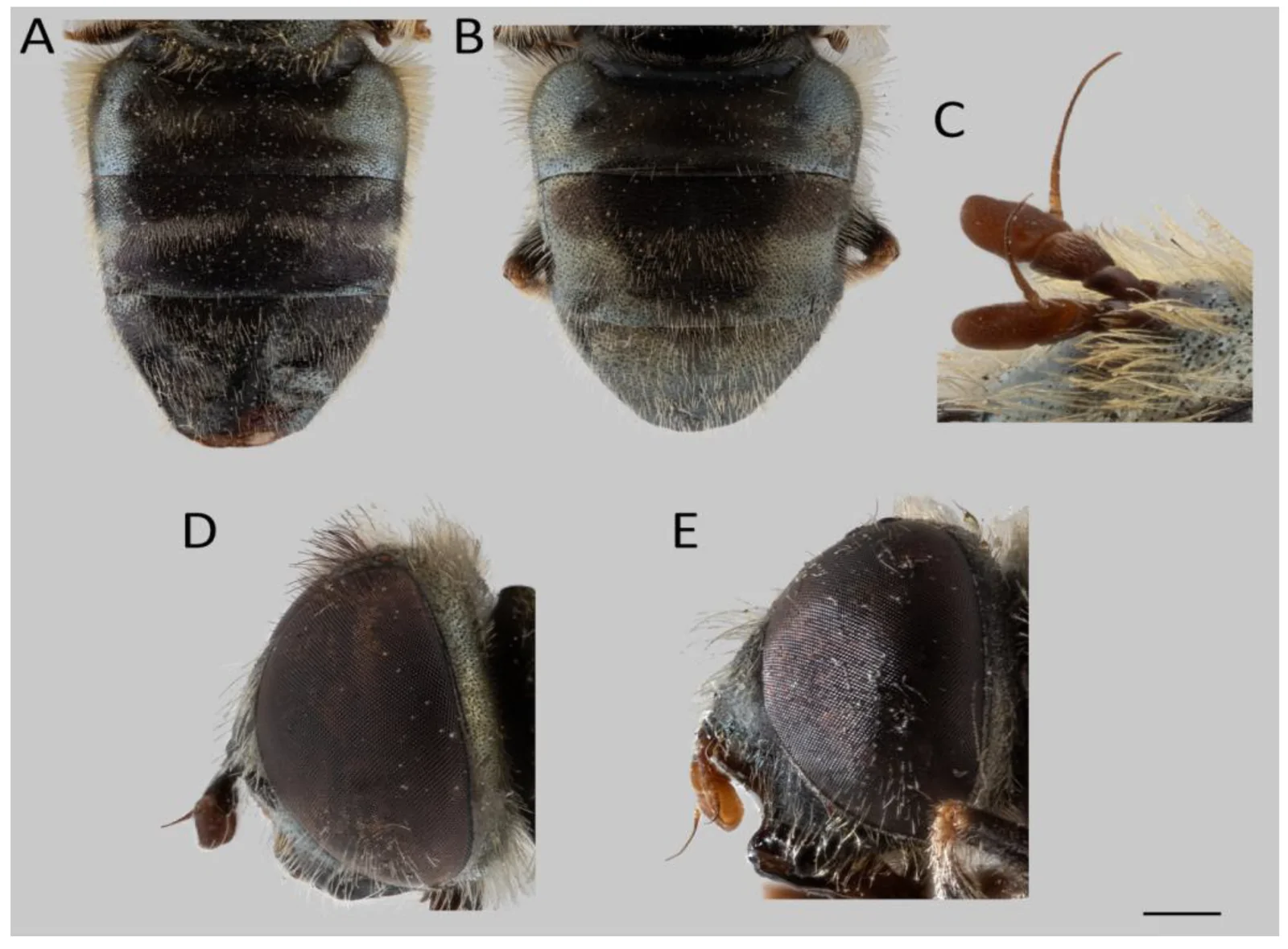
Males of (**A**–**D**) *Merodon syriacus*, (**E**) *M. nasicus*. (**A**,**B**) Abdomen, dorsal; (**C**) antenna, lateral; (**D**,**E**) head, lateral. Scale bar: (**A**,**B**,**D**,**E**) 1 mm, (**C**) 0.75 mm.

**Figure 12:**
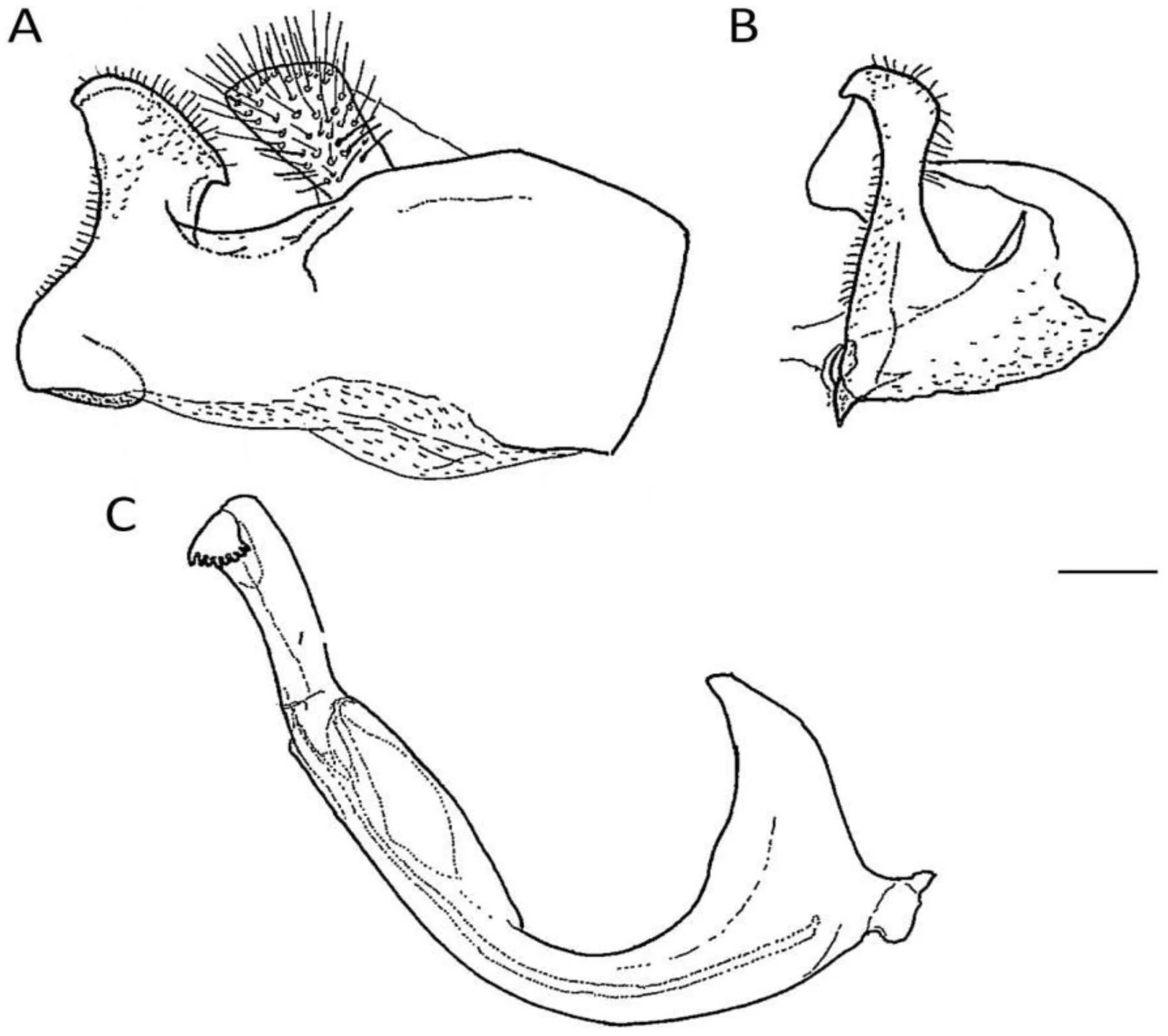
Male genitalia *Merodon syriacus*. (**A**) Epandrium, lateral; (**B**) epandrium, ventral; (**C**) hypandrium, lateral. Scale bar: 0.5 mm.

Species belonging to this subgenus are characterized by the following, most distinct morphological features: stocky abdomen (Figure 11A), the posterior side of mesocoxa covered with pili, the anterior surstylar lobe is undeveloped (Figure 12) and the lateral sclerite of aedeagus is partly or completely reduced. They are divided into five species groups, namely, *aureus*, *bombiformis*, *funestus*, *nanus* and *spinitarsis*, with one unplaced species, *Merodon unguicornis* Strobl, 1909 [24].

*Merodon syriacus* Paramonov, 1929

Merodon pedinum by Hurkmans (unpublished)

Note. Part of the museum material was labeled as Merodon pedinum by Hurkmans. This name was never formally published; however, specimens bearing this label can still be found in some collections.

Type material examined:

*Merodon syriacus*, holotype (studied): “*Merodon syriacus* n. sp.” Paramonov (Paramonow), 1929: 186 (=184). Labels: [N 338]; [Syria]; [*Merodon*/*syriacus*/n. sp. # Typus/Paramonov det.] (SIZK).

Additional material examined:

ISRAEL, Har Meron Reserve, En Zeved, 32.9833 35.4333, 24.iv.2002, 3♀, Freidberg A. leg. (FSUNS ID 04897, 04899, 04920) (TAU); Mt. Meiron, 1100 m asl, 30.iv.1981, 1♀, Freidberg A. leg., det. as *M. chalybeatus* by Hurkmans (FSUNS ID 04930) (TAU); Tivon, 2.iv.1975, 2♂, Kaplan F. leg., det. as M. pedinum by Hurkmans (FSUNS ID 04919, 04918) (TAU); Qatzrin, 4.iv.1987, 1♀, Nussbaum I. leg. (FSUNS ID 04921) (TAU); Gaton, 17.iii.1973, 1♀, Kaplan M. leg., det. as M. pedinum by Hurkmans (FSUNS ID 04929) (TAU); Paar Cave, near Sasa, 33.0333 35.3833, 800 m asl,26.v.2009, 1♂, Freidberg A. leg. (FSUNS ID 04928) (TAU); Jerusalem, Ma’ale Hachamisha, 32.74166 35.046387, 31.iii.74, 2♂ (RMNH); HaDarom, Hava, 30.7166667 34.9166667, 23.iii.1974, 1♀ (RMNH); Jerusalim Kivia Hissmel, Zichron, 18.x.1932, 1♂, dr Rivnay E. leg., det. as *Merodon syriacus* by A. Vujić (RMNH); Jerusalem, Beit HaKerem, 31.7833333 35.2, 17.iii.1951, 2♀, det. as *Merodon lydicus* by Hurkmans, and as *Lampetia hirta* by P. H. Doesburg (FSUNS ID 03323, 03324) (RMNH); Kfar Ezion, 16.x.1935, 1♀, det. as *Lampetia hirta* by P.H. Doesburg and as *Merodon syriacus* by A. Vujić (FSUNS ID 03318) (RMNH); Germak, 15.iv.1962, 1♂, Shapira O. leg. (TAU); Beit HaKerem Wadi Ruaz, 31.7833333 35.2, 17.iii.1951, 3♂ 1♀, Theodor O. leg., det. as *Lampetia hirta* by P. H. Doesburg and *Merodon hirtus* by Hurkmans (RMNH); Beit HaKerem Wadi Ruaz, 31.7833333 35.2, 10.iii.1951, 2♂ 2♀, Theodor O. leg., det. as *Lampetia hirta* by P. H. Doesburg and *Merodon hirtus* by Hurkmans (RMNH); Beit HaKerem Wadi Ruaz, 31.7833333 35.2, 24.iii.1951, 1♂ 1♀, Theodor O. leg., det. as *Lampetia hirta* by P. H. Doesburg and *Merodon hirtus* by Hurkmans (RMNH); Beit HaKerem Wadi Ruaz, 31.7833333 35.2, 29.iii.1952, 1♂, Theodor O. leg., det. as *Lampetia hirta* by P. H. Doesburg and *Merodon hirtus* by Hurkmans (RMNH); Beit HaKerem Wadi Ruaz, 31.7833333 35.2, 22–28.vii.1961, 1♂, Theodor O. leg., det. as *Lampetia hirta* by P. H. Doesburg and *Merodon hirtus* by Hurkmans (RMNH); Beit HaKerem Wadi Ruaz, 31.7833333 35.2, 15.iv.1953, 1♂, Costa M. leg., det. as *Lampetia hirta* by P. H. Doesburg and *Merodon hirtus* by Hurkmans (RMNH); Beit HaKerem Wadi Ruaz, 31.7833333 35.2, 01.iv.1953, 1♀, Theodor O. leg., det. as *Lampetia hirta* by P. H. Doesburg and *Merodon hirtus* by Hurkmans (RMNH); Jerusalim, Kivia Hissmel, 01.iii.1936, 1♂, det. as *Lampetia hirta* by P. H. Doesburg and *Merodon hirtus* by Hurkmans (RMNH); Ma’ale Hachamicha, 31.iii.1974, 2♂ 1♀, Nachbar F. leg., det. as Merodon pedinum by Hurkmans (TAU); Ma’ale Hachamicha, 30.iii.1974, 1♂, Nachbar F. leg., det. as Merodon pedinum by Hurkmans (TAU); Mt. Meron, 1100 m asl, 27.iv.1984, 1♂, Freidberg A. leg. (TAU); Pa`ar Cave near Sasa, 33.0333 35.3833, 800 m asl, 26.v.2009, 1♂, Friedberg A. leg. (TAU); Har Meron reserve, en Zeved, 32.9833 35.4333, 24.iv.2002, 1♂ 2♀, Friedberg A. leg. (TAU); Har Meron reserve, en Zeved, 32.9833 35.4333, 24.iv.2002, 2♂, Friedberg A. and Friedman L. leg. (TAU); Mt. Meron, 1100 m asl, 23.iv.1973, 1♂, Kaplan M. leg., det. as Merodon pedinum by Hurkmans (TAU); Jerusalem, 20.iii.1962, 1♀ (TAU); Jerusalim, Bet Hakerem, 18.iii.1954, 1♂, Theodor O. leg., det. as Merodon pedinum by Hurkmans (TAU); Arbel, 17.iv.1992, 1♀, Friedberg A. leg., det. as *Merodon chalybeatus* by Hurkmans (TAU); Ga`aton, 17.iii.1973, 1♀, Kaplan M. leg., det. as Merodon pedinum by Hurkmans (TAU); J. Jermak, 16.iv.1963, 1♂ 1♀, Margalit J. leg. (TAU); J. Jermak, 15.iv.1962, 1♂, Margalit J. leg. (TAU); Jerusalem, 15.iv.1953, 1♀, Costa M. leg., det. as Merodon pedinum by Hurkmans (TAU); Qaztin, 09.iv.1987, 1♂, Nussbaum I. leg. (TAU); Tivon, 03.iv.1973, 3♂, Nachbar F. leg., det. as Merodon pedinum by Hurkmans (TAU); Tivon, 02.iv.1975, 3♂ 1♀, Kaplan F. and Kaplan M. leg., det as Merodon pedinum by Hurkmans (TAU); Jerusalem, Beit HaKerem, 31.78333 35.2, 20.iv.1953, 1♂, det. as *Merodon hirtus* by Hurkmans and as *Lampetia hirta* by P. H. van Doesburg (FSUNS ID 03319) (TAU); Mount Scopus, Zichron, 15.vii.1930, 1♀, det. as *Merodon syriacus* by A. Vujić and as *Lampetia hirta* by P. H. van Doesburg (RMNH); Wadi Ruaz, Bethakerem, Jerusalem, 20.iv.1953, 1♂ 1♀, Theodor O. leg., det. as Merodon pedinum by Hurkmans (TAU); Jermak, 16.iv.1963, 1♀, Mangalit leg. (FSUNS ID 04931) (TAU); 02.iv.1930, 1♀, det. as *Merodon aeneus* by Hurkmans and as *Lampetia aenea* by Sack (FSUNS ID 04352) (BMNH); TÜRKIYE, Icil Tasucu, 10 km SW Silifke, 36.3777778 33.9344444, 300m asl, 14.iii.1984, 1♀, J.A.W. Lucas leg., det. as *M. syriacus* by Vujić in 2008 (FSUNS ID 03322) (RMNH); Icil Tasucu, 10 km SW Silifke, 36.3777778 33.9344444, 300 m asl, 14.iii.1984, 1♂, J.A.W. Lucas leg., det. as *M. syriacus* by Vujić in 2008 (FSUNS ID 03320) (RMNH); Icel, Icil Tasucu, Silifke, 36.3777 33.9344, 100–300 m asl, 15.iii.1984, 1♂ (FSUNS ID 03321) (RMNH); Icel, Icil Tasucu, Silifke, 36.3777 33.9344, 300 m asl, 14.iii.1984, 1♀ (FSUNS ID 03343) (RMNH); Icel, Tasucu, 10 km SW from Silifke, 36.3269444 33.89111, 14–17.iii.1984, 13♂ 7♀ (RMNH); Nigde, Nigde, 37.8333 34.75, 08.v.1961, 2♀ (RMNH); near Mugla, 37.19513 28.35198, 693 m asl, 06.iii.2014, unk. sex, Vujić A. and Ačanski J. leg. (FSUNS ID 06836) (FSUNS); Icel, Icil Tasucu, Silifke, 36.3777 33.93444, 300 m asl, 14.iii.1984, 1♂ 1♀ (FSUNS ID 03320, 03322) (RMNH).

**Figure 13:**
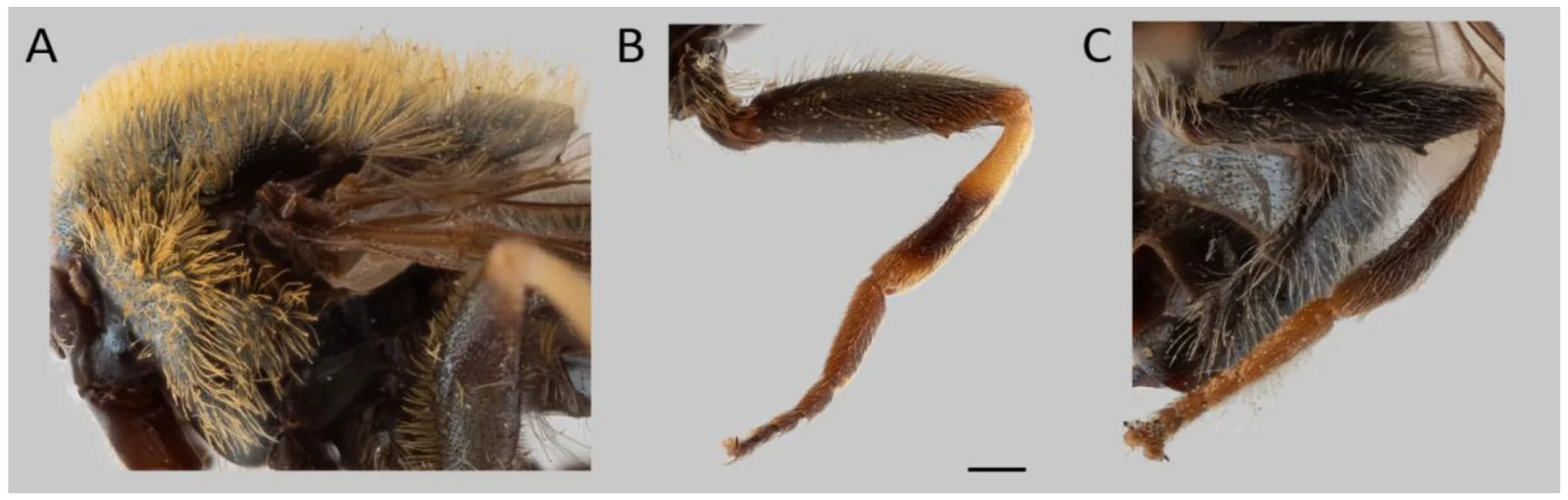
Males of (**A**,**B**) *Merodon syriacus*, (**C**) *M. nasicus*. A Scutum, dorsal; (**B**,**C**) metaleg, anterior. Scale bar: (**A**) 0.75 mm and (**B**,**C**) 1 mm.

Diagnosis: Small (6–8 mm), black species with or in some males without narrow, white pruinose fasciae on tergites (Figure 11A,B). Pedicel elongated, almost as long as basoflagellomere (Figure 11C). Oral margin slightly protruded (Figure 11D). Face and frons shiny, without or with weak pruinosity. Scutum covered with yellow pili and few black pili near the wing base (Figure 13A). Metatrochanter smooth, without calcar (Figure 13B). Metafemur with small, less dentate apicoventral triangular lamina (Figure 13B). Male genitalia with angular posterior surstylar lobe, dorsally slightly hook-like from ventral view (Figure 12B); hypandrium narrow (Figure 12C). Most similar to East African species *M. nasicus* Bezzi, 1915, which it differs by a less protruded oral margin (Figure 11D) (distinctly protruded in *M. nasicus* (Figure 11E)), and is determined by presence of black pili on scutum near the wing base (Figure 13A) (absent in *M. nasicus*), and in small, less dentate apicoventral triangular lamina on metafemur (Figure 13B) (distinctly dentate in *M. nasicus* (Figure 13C)), as well as by male genitalia features (in *M. syriacus* posterior surstyle lobe almost straight with slightly widened apical part (Figure 12A) and in *M. nasicus* S-shaped (Figure 19D in Vujić et al. [32])).

Redescription.

Note. The original description without illustrations was based on one male from Syria [48].

Length: Body 6–8 mm and wing 5–7 mm (*n* =12).

Male: Head: Antenna reddish-brown; pedicel slightly elongated (Figure 11C) (relation of scape: pedicel: basoflagellomere = 1: : 2.5); basoflagellomere concave dorsally, with acute apex; arista reddish-brown, thickened basally, 2.5 times as long as basoflagellomere. Face black, without or with weak pruinosity, covered with yellowish pili, except on bare medial vitta that occupies about ⅓ of width of face. Oral margin slightly protruded (Figure 11D). Frons black, without gray pruinosity, covered with yellowish pili. Vertical triangle isosceles, predominantly covered with long, yellow pili. Ocellar triangle isosceles. In the upper half of the eye pili are black and in the lower part white to grayish, as long as scape. Eye contiguity about 14–16 ommatidia long. Occiput black, partly pruinose, covered with yellowish pili.

Thorax: The mesonotum dark brown, covered with yellowish pili, except few black pili near wing basis (Figure 13A); scutum without or with indistinct pruinose longitudinal vittae. Pleura dark brown, shiny, with indistinct gray pruinosity on the parts covered with yellow pili: anterior part of proepimeron, posterior part of anterior anepisternum, most of the posterior anepisternum except anterior part, anteroventral and posterodorsal parts of katepisternum, anepimeron and metasternum. Wing hyaline, mostly covered with microtrichia; veins yellow to brown. Calypter pale yellow. Haltere dark brown. All three femora are dark brown to black, except reddish apex; tibiae mostly reddish, medially dark brown (Figure 13B). Metatrochanter without calcar. Metafemur moderately thickened, with less developed apicoventral triangular lamina, only slightly dentate (Figure 13B). Pili on legs predominantly whitish to yellow, except few long black pili in the apical part of mesofemur.

Abdomen (Figure 11A,B): Dark brown, covered with yellow to whitish-yellow pili laterally. Tergum 1 covered with short whitish pili; tergum 2 usually with narrow medial, pruinose fascia, interrupted in the middle, and covered with yellowish pili laterally and medially, but mostly covered with short black pili; terga 3 and 4 with or without narrow medial, pruinose fasciae, interrupted in the middle; tergum 3 covered with short black pili medially; tergum 4 covered with longer whitish to yellowish pili, except few black pili in anterior half. Sterna brown, pruinose, covered with white or yellowish pili.

Male genitalia: Posterior surstylar lobe angular (Figure 12A), dorsally slightly hook-like from ventral view (Figure 12B); anterior surstylar lobe undeveloped (Figure 12A); cercus square-like (Figure 12A). Hypandrium sickle-shaped, with very narrow theca (Figure 12C).

**Figure 14:**
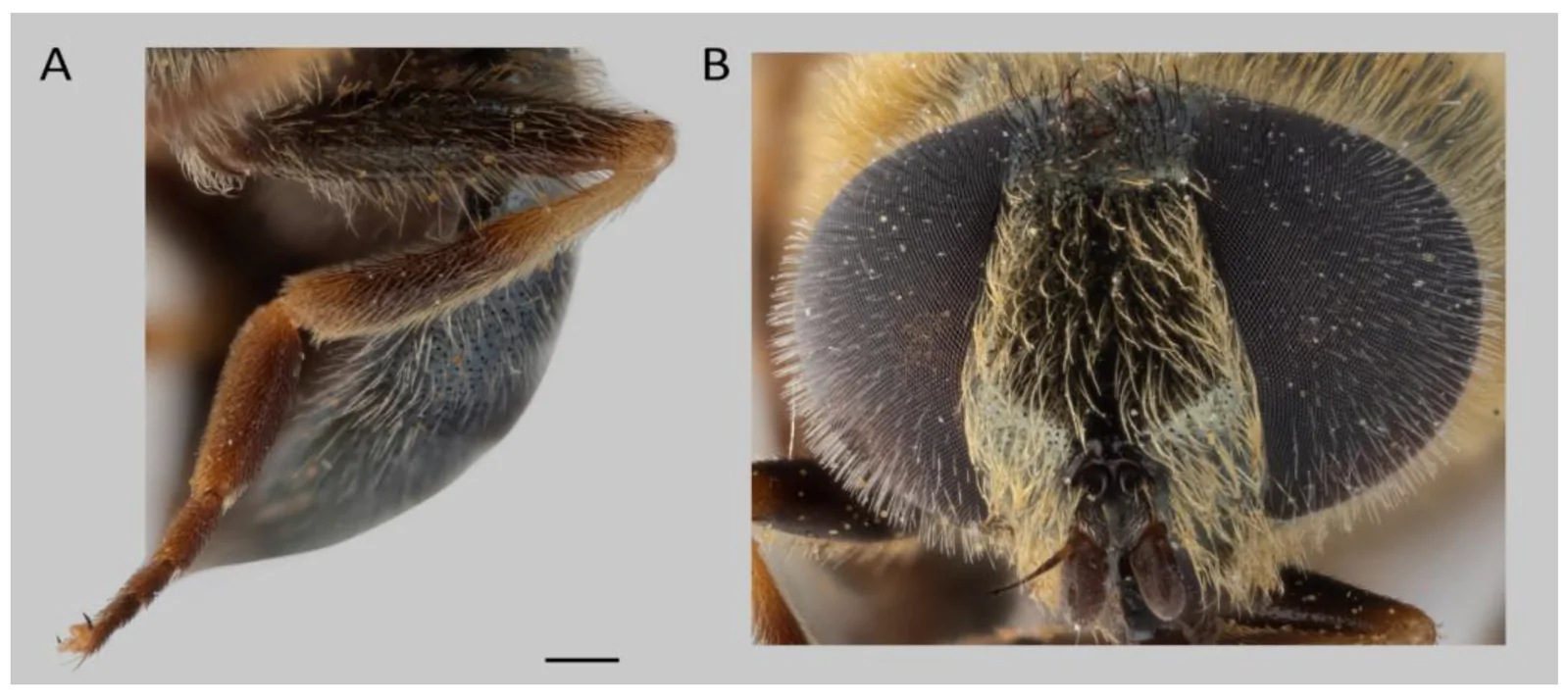
Female of *Merodon syriacus*. (**A**) Metaleg, anterior; (**B**) head, frontal. Scale bar: (**A**) 1 mm and (**B**) 0.5 mm.

Female: Similar to the male except for usual sexual dimorphism: metafemur usually with very small dens on triangular lamina (Figure 14A); frons shiny (Figure 14B); vertex shiny, mostly covered with black pili.

**Figure 15:**
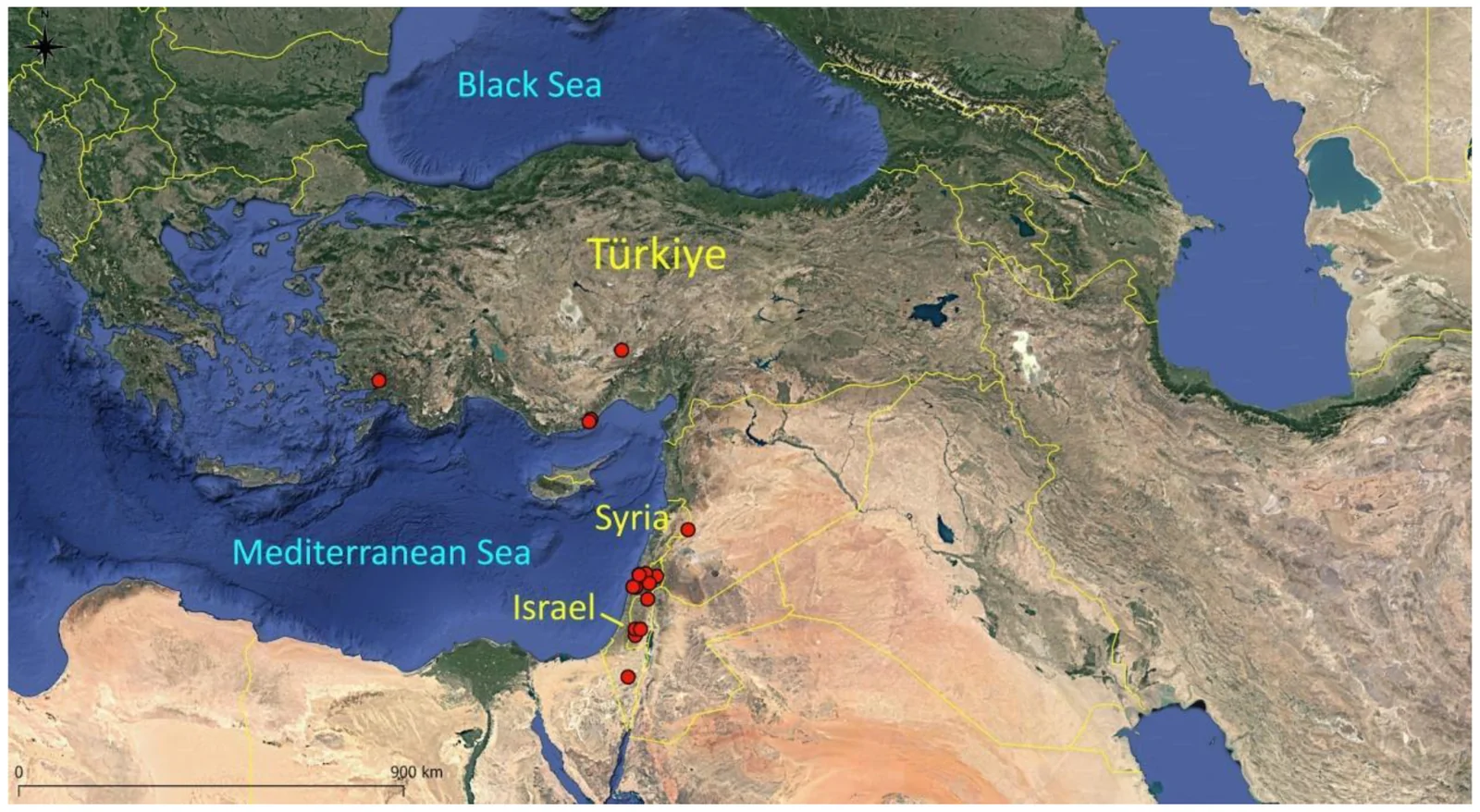
Distribution map of *Merodon syriacus*.

Distribution: This species is registered in Syria, Israel and Türkiye (Figure 15).

### 3.4. Molecular Evidence

**Figure 16:**
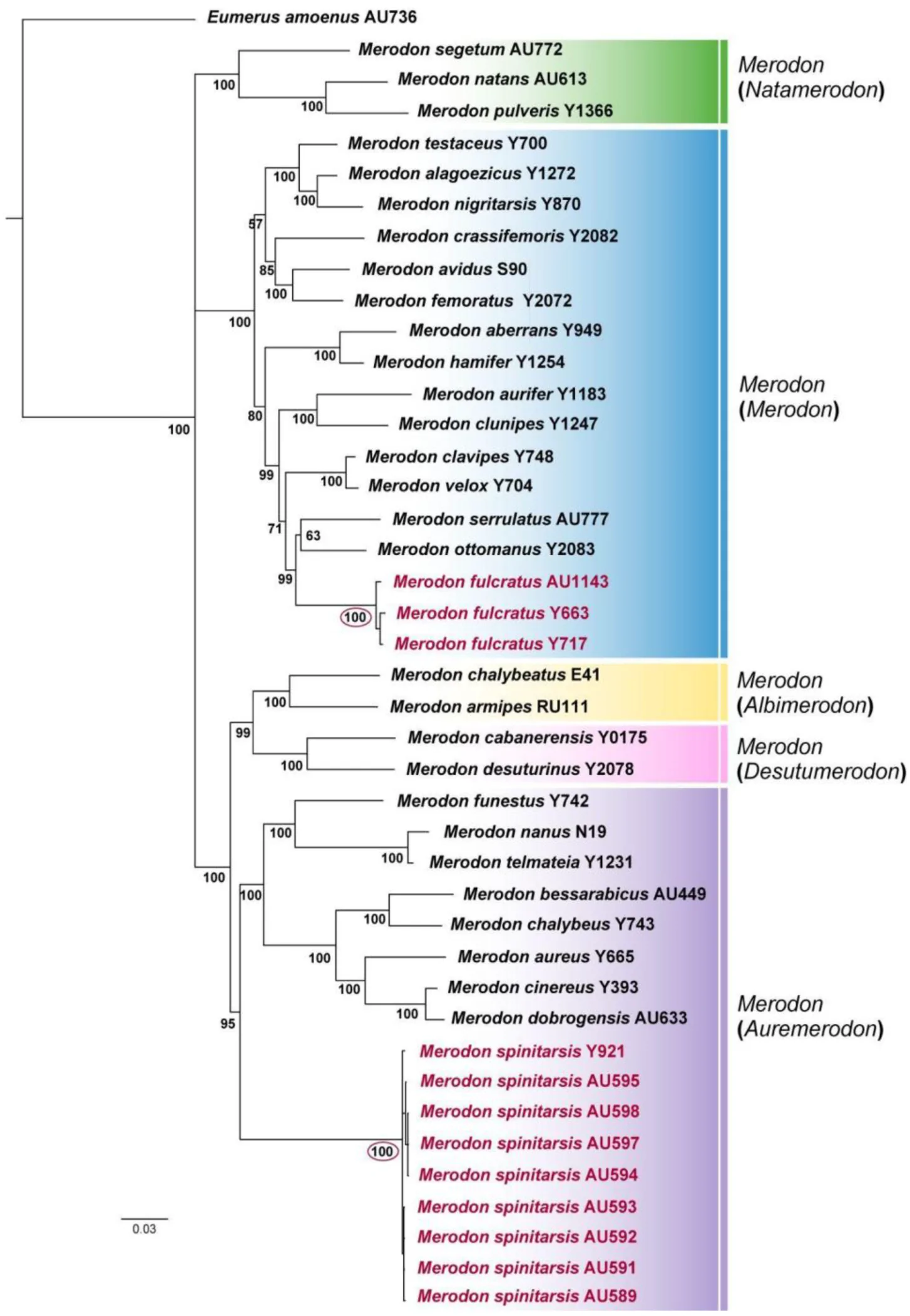
The Bayesian inference phylogenetic tree of genus *Merodon* based on the combined dataset (*COI* gene + *28S rRNA* gene sequences), with Bayesian posterior probabilities indicated near nodes.

In order to clarify the systematic placement of the *Merodon fulcratus* and *M. spinitarsis* groups, a Bayesian phylogenetic analysis was conducted using a concatenated two-gene dataset (mtDNA *COI* and nuclear *28S rRNA*). The resulting tree (Figure 16) strongly supports the previously defined subgenera in Veselić et al., 2025 [24]. Despite a significant intraspecific variability in the morphological characters, all analyzed specimens of *M. fulcratus* are robustly placed within *M.* (*Merodon*) (posterior probability, PP = 100). Likewise, the clade comprising all analyzed specimens of *M. spinitarsis* is strongly supported, forming a distinct lineage within *M.* (*Auremerodon*) (PP = 100). The sequences of *Merodon complicatus* sp. n. and *M. syriacus* were unavailable and therefore these species were not included in the molecular analysis.

## 4. Discussion

### 4.1. Morphological and Molecular Aspects

*Merodon* (*Merodon*) consists of 10 species groups and 9 unplaced species [24]. Numerous recent revisions have resolved the status of species within most of these groups [7,10,11,13,14,15,21], with the exception of the *fulcratus* and *italicus* species groups. One of the results of this paper is a taxonomic revision of the *fulcratus* group, which is defined by a very unique character within the entire genus *Merodon*: complete separation of the eyes in males (dichoptic). This feature occurs in only one other species, *M. flavocerus* Hurkmans, 2018, from *M.* (*Desutumerodon*), but is less pronounced [49]. The two valid species that make up this group are *M. dichopticus* and *M. fulcratus*, where males are clearly distinguished by the shape of the male genitalia, whereas reliable morphological characteristics for separating females have not yet been found. Since the distributions of the two species do not overlap, females can currently be identified based on the locality where the males are found or using genetic data in the future. Although significant variability in the shape of the posterior surstylar lobe has been observed within *M. fulcratus*, the inferred phylogenetic topology resolved all analyzed specimens into a highly supported clade, confirming the status of *M. fulcratus* as a unique species within *M.* (*Merodon*).

Of the five species groups that make up *M.* (*Auremerodon*), the *spinitarsis* group remains unrevised. This paper defined the *spinitarsis* species group based on key morphological characteristics relating to the structure of the male genitalia: a strongly modified hypandrium with stitched theca and a pointed posterior surstylar lobe. Based on the reviewed material, slight variability was observed in the prominence of the concavity at the margin between the anterior and posterior surstylar lobes and in the width of the apical part of the theca of the hypandrium; however, all these variant forms are present in both species, and no additional differences were observed in the shape of the male genitalia when comparing *M. complicatus* sp. n. and *M. spinitarsis*. Such intraspecific variability is not uncommon among valid species of the genus *Merodon*, and has been documented, for example, in *Merodon serrulatus* (Wiedemann in Meigen, 1822) [10], *M. constans* (Rossi, 1794) [9], *M. calcaratus* (Fabricius, 1794) [12] and *M. fulcratus*, presented above. On the other hand, diagnostic characteristics for distinguishing females of the *spinitarsis* species group from those of other groups are related to the appearance of the terga and the color of the tibiae and tarsi. However, females of the two valid species within this species group do not show any morphological differences, which is why, as with the previous group, they are separated solely on the basis of distribution: *M. complicatus* is present in Israel and *M. spinitarsis* in Bulgaria, Greece, Romania and Türkiye. According to the molecular dataset, *M. spinitarsis* has been resolved as a highly supported clade within *M.* (*Auremerodon*), confirming the phylogenetic affinity and placement of this unique *Merodon* species. In Veselić et al. [24], molecular data analyses (both parsimony and maximum likelihood) showed that *M. spinitarsis* clusters with *M. bombiformis* Hull, 1944, a result the authors attributed to the long-branch attraction phenomenon.

The concept of *M. syriacus* used by different authors has been incorrectly interpreted and applied to the species described here as *M. complicatus* sp. n., a member of the *spinitarsis* species group (as in [17,50,51]). Examination of the holotype of *M. syriacus* clearly shows that it is most similar to the East African species *M. nasicus* Bezzi, 1915, from the *bombiformis* species group. However, in the absence of genetic support, it should be considered an independent entity within *M.* (*Auremerodon*). Veselić et al. [24] included *M. syriacus* in a phylogenetic analysis of the genus *Merodon* under the name *M. crassicornis* Sack, 1913. Since sequence data for this species were unavailable, it was included in the parsimony and maximum likelihood analyses based solely on the morphological character matrix. In both resulting trees, it was placed within the *aureus* species group with bootstrap support exceeding 85, together with *Merodon aureus* Fabricius, 1805*; Merodon bessarabicus* Paramonov, 1924; *Merodon cinereus* (Fabricius, 1794); *Merodon dobrogensis* Bradescu, 1982; *Merodon minutus* Strobl, 1893; *Merodon pumilus* Macquart, 1849; *Merodon puniceus* Vujić, Radenković & Pérez-Bañón; and *M. unguicornis*; however, it did not show a clear affiliation with any particular subgroup [24]. The status of *M. crassicornis* is still unresolved due to the lost type material, but the presence of a strongly dentate apico ventral triangular lamina in this species (listed in the original description) undoubtedly indicates that this species is not conspecific with *M. syriacus*.

### 4.2. Distribution

The two *Merodon* species groups revised in the present study are distributed in the north-western and central parts of the Middle East. The distribution area of the *fulcratus* group extends from northern Türkiye, Georgia and southern Turkmenistan, across Iran, to Israel in the south-west. Considering the two valid species recognized within the group, *M. fulcratus* has a more eastern distribution, occurring throughout Iran to Turkmenistan within the Kopet Dag area, while *M. dichopticus* reaches the western parts of the area. The latter species is found across the Anatolian Peninsula and south-western parts of the Levant region, with records from Georgia and northern Iraq. The southernmost part of the distribution area of the *spinitarsis* species group includes Israel, where *M. complicatus* sp. n. has so far only been recorded; most records are from northern areas, i.e., Mount Hermon, Meron and Carmel, with several findings in central Israel. A further species from the group, *M. spinitarsis*, has a larger and more fragmented distributional range, spanning throughout Türkiye and across Bulgaria to southern Romania in the north and Greece to the west. Another species, *M. syriacus*, considered an independent entity within *M.* (*Auremerodon*), has been recorded in the Levant region and southern Anatolian Peninsula.

The distribution ranges of *Merodon* taxa analyzed in the present study are mostly located within the three (out of the world’s 36) biodiversity hotspots in Europe and West Asia [2,52], which are among the most biologically diverse terrestrial areas, yet are threatened and understudied in terms of their invertebrate fauna. Namely, the *fulcratus* species group is mostly within the Irano-Anatolian biodiversity hotspot and partly within the Caucasus biodiversity hotspot, while the *spinitarsis* group and *M. syriacus* occur within the eastern part of the Mediterranean Basin biodiversity hotspot. These topographically complex areas, characterized by intense orogenic activity and rich geological history, with geomorphic, altitudinal and climatic variation, and thus short-distance changes in ecological factors and habitats, have provided barriers favoring isolation and speciation, and also refugia and corridors fostering species dispersal [16,17,19,21,22].

Despite the high diversity of its biota and the often severe threats to its environment, the capacity of biodiversity-related research has long been considered inadequate in most parts of the Middle East [53]. Located at the junction of continents and acting as a transition zone between biogeographic regions, in recent years this center of speciation has been recognized for its unique biological diversity including hoverflies (e.g., [20]). However, some regions have received less attention than others in terms of hoverfly faunistic research. For example, Iran has been one of the underexplored regions, lacking long-term systematic studies, but in recent years it has proven to host a significant diversity of *Merodon* species [13,14,15,20,21,22,54,55,56]. Nevertheless, the number of hoverfly species in different provinces varies, being related not to their area size but rather to the intensity of the studies conducted; although Iran’s provinces have different climates and vegetation, it seems that more research could reveal a richer hoverfly fauna throughout this country [23]. Some regions, such as the Caucasus with several endemic *Merodon* species known [11], have been of longstanding interest to hoverfly researchers, but the reviews of specific parts of the region have been rare [57]. For example, the Nakhchivan Autonomous Republic of Azerbaijan, the type locality of *M. dichopticus*, has been reviewed only recently, describing one new and reporting several more *Merodon* species as new records [58]. Even in countries with many hoverfly faunistic studies in recent decades, such as Türkiye, targeted investigations in some regions and provinces remain quite limited, and new records of hoverfly taxa including *Merodon* species have been reported recently (e.g., [59,60]). Overall, many new records and species have been provided for the broader region of the Middle East in previous years (e.g., [7,14,15,19,20,21,22,56]), confirming the need for further investigations in order to complete the picture of *Merodon* species distributions within the area. In addition, certain Palaearctic areas such as Central Asia and Pakistan with numerous endemics are potentially significant for understanding the evolution and phylogeny of the genus; however, they have been under-investigated [1,10,21]. Moreover, a recent study has indicated a potential range extension of the genus further south-east into the Oriental biogeographic region, noting that the existing gap in the range may reflect insufficient sampling or ecological constraints across transitional habitats and suspecting possible incidental use of an available host (an introduced cultivated geophyte) and local adaptation to anthropogenic habitats [61].

Since *Merodon* phytophagous larvae feed on underground storage organs of geophytes (e.g., [62,63]), the geographical distributions of *Merodon* taxa have been considered to be linked to the presence of flora rich in these plants [16,19]. Within the Mediterranean region recognized as a center of *Merodon* diversity, Türkiye has the highest species richness and endemism [1,16,17]. The Mediterranean Basin, particularly Anatolia, is also one of the world’s most biodiverse areas in terms of geophytes [64,65], the Irano-Anatolian hotspot is one of the global centers of floral diversity and endemism, and a high percentage of the Caucasus hotspot’s numerous plant species are restricted to the region [2,66]. However, data on *Merodon* developmental stages are very scarce, and thus far descriptions of larvae and/or puparia have been published for only 17 species [67,68], while the most recent review of proven natural interactions between larvae of *Merodon* and their host plants listed 27 *Merodon* species [69]. This comprehensive study by Popov et al. [69] confirms the natural-history unity of the *M. constans* species group through monophagy on Galantheae (Amaryllidaceae) host plants, noting the conservation implications of close associations of *Merodon* species with certain plant species. The need for further research of the relations of *Merodon* taxa and their larval hosts is important due to the potential risks to host-plant availability [22]. The primary threats leading to habitat loss in the investigated areas include agricultural intensification and infrastructure development, and also forest fires and pollution in the Mediterranean Basin, illegal logging in the Caucasus, and deforestation, mining, and military operations in the Irano-Anatolian biodiversity hotspot [2]. Apart from habitat destruction, many geophyte plants have high ornamental value and are further affected by anthropogenic pressure from uncontrolled commercial harvesting for the horticultural trade in, e.g., Türkiye [64] and Iran [66].

These issues underline the need for conservation efforts and for further broadening of systematic studies of hoverfly fauna in these areas, including both field research and detailed revisions of available museum and other collections, since the diversity is potentially higher than has been recognized so far. Cases of a lower number of *Merodon* species in some countries compared with neighboring ones, often smaller in area, probably do not reflect a poorer fauna but rather insufficient faunistic surveys [11,23]. By clarifying the nomenclatural issues and the status of certain species, the present study contributes to improving our knowledge of the *Merodon* taxonomy and distribution in the Middle East and to understanding the significance of underexplored regions, especially biodiversity hotspots, with the potential for high and often hidden diversity within one of the most species-rich genera of hoverflies.

## Data Availability

All data supporting the findings of this study are provided in the main text and the accompanying Supplementary Information. The molecular sequence data generated and used in this study are publicly available through the GenBank database at https://www.ncbi.nlm.nih.gov/genbank/ (accessed on 1 October 2026).

## Supplementary Materials

The following supporting information can be downloaded at https://www.mdpi.com/article/10.3390/insects17101078/s1: Table S1: List of specimens used in molecular analysis with additional information and GenBank accession numbers. Table S2: Detailed data related to the studied specimens of *Merodon spinitarsis.*

## Abbreviations

The following abbreviations for museums and private entomological collections are used in this manuscript: BM coll. (Bartak Miroslav collection, Czech Republic); BMNH (Natural History Museum, London, UK); FSUNS (Faculty of Sciences, University of Novi Sad, Serbia); HJ coll. (Hadrava Jiří collection, Czech Republic); KCZ coll. (Khosravijan Chatroodi Zoerh collection, Iran); KS coll. (Khaghaninia Samad collection, Iran); MNB (Museum für Naturkunde, Berlin, Germany); NMPC (National Museum Prague, Prague, Czech Republic); RM coll. (Reemer Menno collection, the Netherlands); RMNH (Naturalis Biodiversity Center, Leiden, the Netherlands); SIZK (I.I. Schmalhausen Institute of Zoology, National Academy of Sciences, Kiev, Ukraine); SZMN (The Siberian Zoological Museum of the Institute of Systematics and Ecology of Animal Siberian Branch of the Russian Academy of Sciences, Novosibirsk, Russia); TAU (Tel Aviv University, Tel Aviv, Israel); ZHMB (Zoologisches Museum of the Humboldt University, Berlin, Germany); ZISP (Zoological Institute, Russian Academy of Sciences, St. Petersburg, Russia).
